# Selection of Nonlethal Early Biomarkers to Predict Gilthead Seabream (*Sparus aurata*) Growth

**DOI:** 10.1155/anu/9918595

**Published:** 2025-01-06

**Authors:** Rafael Angelakopoulos, Andreas Tsipourlianos, Katerina A. Moutou, Alexia E. Fytsili, Anthi Tsingene, Eleni Galliopoulou, Leonidas Papaharisis, Zissis Mamuris, Themistoklis Giannoulis, Arkadios Dimitroglou

**Affiliations:** ^1^Department of Biochemistry and Biotechnology, Laboratory of Genetics, Comparative and Evolutionary Biology, University of Thessaly, Biopolis 41500, Larissa, Greece; ^2^Department of Research and Development, Avramar Aquaculture SA, Athens, Greece; ^3^Department of Animal Science, Laboratory of Biology, Genetics and Bioinformatics, University of Thessaly, Gaiopolis 41334, Larissa, Greece; ^4^Department of Animal Science, Laboratory of Applied Hydrobiology, Agricultural University of Athens, Iera Odos 75 11855, Athens, Greece

**Keywords:** biochemical markers, growth potential, nonlethal markers, transcriptome markers

## Abstract

One of the main challenges in aquaculture is the constant search for sustainable alternative feed ingredients that can successfully replace fishmeal (FM) without any negative effects on fish growth and health. The goal of the present study was to develop a toolbox for rapidly anticipating the dynamics of fish growth following the introduction of a new feed; nonlethal, biochemical, and molecular markers that provide insights into physiological changes in the fish. A nutritional challenge by feeding a conventional feed rich in FM protein (FM diet) versus an experimental feed rich in plant protein (PP) and low FM inclusion (PP diet), in 20 different families of gilthead sea bream (*Sparus aurata*) was performed. Fifteen and 30 days after the initiation of the nutritional challenge, the transcriptional response of gilthead seabream erythrocytes along with classical hematological biochemical markers were compared. Zootechnical, biochemical, and transcriptome data from each family under different dietary treatments were combined into a classification model to identify variables that can predict the growth rate at the end of the 14-month farming period (July 2018–September 2019). A highly accurate model was produced (*A* > 80%) based on the combination of seven markers (five molecular and two biochemical markers) and with high potential in separating faster and slower growing fish as early as 30 days after the initiation of feeding.

## 1. Introduction

The aquaculture sector faces a major challenge to sustainably meet the increasing consumers' demands, which are expected to double by 2030 [1]. The constantly growing aquaculture sector is driving a rising need for fishmeal (FM) and fish oil [2, 3]. Overexploitation of fish stocks must always be avoided to ensure sustainable management while exploring alternative ways to meet the growing demand for marine feed ingredients tonnage sourced from fisheries [1]. As a result, developing novel feed strategies using sustainable resources to replace FM and fish oil poses a significant challenge to nutritionists, feed industry, and researchers worldwide; searching for highly nutritive, sustainable, and cost-effective raw materials without compromising fish welfare [4–6]. Plant proteins (PPs) are considered to be sustainable alternative protein sources to FM in fish feed production and their use has been expanding over the last decade [7, 8]. However, the presence of a variety of endogenous antinutritional factors and a high nondigestible carbohydrate content in PPs can compromise fish physiology and metabolism [9–11]. This is also reflected on the expression of genes involved in the metabolism of amino acids, lipids, carbohydrates, and immunological processes when switching from fish-based protein to plant-based protein diet [12]. These nutritionally mediated effects can be detrimental to the health and growth of fish.

Numerous researchers focus on the genotype by diet (*G* × *D*) interactions and whether certain individuals can utilize plant-based diets better than others [13–18]. Selecting for a more adaptable response to dietary changes with minimal impact on growth and health has also become one of the objectives of genetic selection in gilthead seabream (*Sparus aurata*) [19, 20], complementing the primary objective to enhance the overall growth rate. The current design of the breeding programs in gilthead seabream is based on the collection of data from phenotypic characteristics during the rearing period, implying that fish must spend over a year in fish cages. This approach not only escalates the breeding program costs and risks but also disregards potential variations in development trajectories or intermediate physiological states [21].

Nonlethal, early detection markers that can be determined without sacrificing the fish have substantial advantages and are in line with fish welfare framework [22]. The selection of peripheral blood is an accessible source of data toward this direction. Blood sampling is simple, and it enables the analysis of alterations in the physiological status [23]. Recent studies have demonstrated that the peripheral blood cell transcriptome is an essential tool for detecting physiological changes in response to different stimuli, for effective genetic selection [24, 25]. Peripheral blood cell transcriptome analysis offers several advantages, including the ability to monitor nonlethally the gene expression profiles reflective of the fish health and environmental conditions. Identifying biomarkers associated with stress responses, disease resistance, and growth rates to inform breeding programs and health management strategies has been revolutionary in aquaculture. The nonlethal nature of blood sampling aligns with ethical and welfare considerations, enabling repeated measures for longitudinal studies and providing comprehensive insights into the dynamic physiological changes in fish. Therefore, whole blood transcriptome analysis is a promising approach that can provide essential data for identifying potential relationships between blood traits and production traits as well as for developing conveniently accessible markers to monitor physiological changes induced by factors, such as feed [26, 27].

The goal of the study was to develop early, highly sensitive, biochemical, and genetic markers indicative of the nutritional status of gilthead seabream. Hence, the response of gilthead seabream 15 and 30 days after nutritional challenge was compared, using a high level of FM inclusion feed versus a challenging feed with high level of PP inclusion, formulated to similar nutritional value.

## 2. Materials and Methods

### 2.1. Ethics Statement

All examined biological materials were derived from fish reared and harvested at commercial farms, registered for aquaculture production in EU countries. Animal sampling followed routine procedures and samples were collected by a qualified staff member from standard production cycles. All experimental procedures have been approved (protocol id 99/24889) by the Departmental Animal Care Committee following the existing Greek (PD 56/2013) and EU (Directive 63/2010) legislation on the care and use of experimental animals.

### 2.2. Rearing Process-Feeding Experiment-Zootechnical Data

Gilthead seabream juveniles originated from the AVRAMAR SA commercial family-based breeding program. All families were produced after artificial insemination between preselected (based on the same selection traits) breeding candidates. After hatch, a commercial rearing protocol was followed, and fish remained in the hatchery until they were individually tagged using a unique intraperitoneal glass RFID tag. When tagging was completed, fish were prepared for transportation in designated commercial sea cages farm in Paleros, Aitoloakarnania, Greece. One thousand (*n* = 1000) fish originating from 20 full-sib families were randomly selected and evenly distributed into two replicate sea cages (4 × 4 × 4 m depth). Fifty individuals from each family were split equally between the two cages, with 25 fish per family allocated to each cage. The trial took place at a commercial production farm following the standard farming procedures of the farm. In order to minimize the burden of the trial on the daily operation of the farm, only two cages were allowed to run the trial. Upon arrival and during the acclimatization period at the sea, fish from both cages were fed the same commercially available feed (Feedus Blue Line 3.5 mm). The feeding trial started after a day of fasting. One sea cage was fed with a FM-based feed (FM diet, average weight: 13.23 ± 2.45 g,) and the other was fed with a PP-based feed (PP diet, average weight: 13.67 ± 2.29 g) of a similar nutritional value. The composition is given in feed percentage (Table 1). Two samplings, 15 (D15) and 30 (D30) days after the initiation of the feeding trial were performed, and all available fish in the cages were sampled. The sampling points were set as early as possible in an attempt to identify biomarkers that respond quickly to nutritional changes.

During the experimental period, fish were fed twice a day until apparent satiation. Feeding was performed by the same two people by changing order every day to minimize the effect of subjectiveness on fish feeding satiation. Daily feed consumption per cage and water temperature were both recorded. Water temperature and oxygen levels throughout the feeding trial are presented in Figure S1.

The two groups were monitored until the fish reached a harvest weight of ~400 g, at which point mortality, weight, and fat content were recorded. Weight measurements were taken in September, November, January, March, July, and August for all available fish. Since all fish were pit-tagged, they were weighed individually throughout the experiment to ensure accurate individual weight monitoring.

Fat content was measured at the end of the trial, using a Distell fat meter FFM-692 (Distell, UK), set up accordingly and based on four measurements on the same side for each fish. Specific growth rate (SGR) for each fish was calculated based on the following formula: %SGR = [(ln (final weight (g)) − ln (initial weight (g))) × 100/*t* (days)].

### 2.3. Blood Sampling

At sampling, all available fish (600) were carefully removed from the sea cages using a net and promptly immersed in an anesthetic (150 ppm ethylene glycol monophenyl ether), to minimize stress during sampling and prevent any movement of the fish. Fish were individually identified based on their unique Pit Tag and blood samples were collected from the caudal vein and immediately centrifuged (6000 *g* for 5 min) on-site to separate the serum from erythrocytes. This approach was adopted to minimize the duration of each sampling step. Subsequently, the fish were transferred to clean water for recovery before being returned to their cages. Erythrocytes were stored with RNAlater (Sigma–Aldrich cat no: R0901) at −20°C and serum was transferred at −80°C until further analysis. Samplings were performed on D15 and D30 after the start of the feeding trial.

### 2.4. Triglycerides Levels in Serum

Triglycerides levels were measured using a colorimetric assay kit according to the manufacturer's protocol (Biosis, cat no: 000244) with minor modifications. Briefly, the volumes were adjusted proportionally for use in a microplate. The measurement was conducted in a microplate using a spectrofluorometer (Varioskan LUX multimode microplate reader, Thermofisher).

### 2.5. Cholesterol Levels in Serum

For the quantification of total cholesterol levels in fish serum a colorimetric assay kit (Biosis, cat no: 001564) was used according to the manufacturer's protocol with minor modifications. Briefly, the volumes were adjusted proportionally for use in a microplate. This colorimetric reaction was measured in a microplate using a spectrofluorometer (Varioskan LUX multimode microplate reader, Thermofisher).

### 2.6. Protein Content in Serum

Serum protein content was measured with the Bradford method [28] using bovine serum albumin as a standard. Two replicates per sample were performed to secure reproducibility.

### 2.7. RNA Isolation and cDNA Synthesis

Total RNA was extracted from erythrocytes using the E.Z.N.A. Total RNA Kit I (OMEGA bio-tek cat no: R6834-02) according to the manufacturer's protocol. In brief, erythrocytes stored in RNAlater were centrifuged to remove the RNAlater and then lysed using a bead-beater (Precellys 24 tissue homogenizer, Bertin instruments). To remove traces of genomic DNA, an extra step of DNAse treatment was performed with DNA-free DNA Removal Kit (Invitrogen cat no: AM1906). RNA quality was evaluated via gel electrophoresis and quantification was made using a microvolume spectrophotometer (Quawell Q3000). Total RNA was stored at −80°C. For the cDNA synthesis, 1 μg of total RNA was reverse transcribed using high-capacity cDNA reverse transcription kit with RNase Inhibitor (Applied Biosystems cat no: 4374966).

### 2.8. RNA Sequencing and Bioinformatics Analysis

Six families were selected for transcriptome analysis based on the observed SGR patterns (see Section 3.1). In total, 24 collective samples (six families × two sampling days × two diets) were created by pooling equimolarly total RNA from 12 fish per family/sampling day/diet, and they were sent to Novogene Europe. The indexed libraries were sequenced on HiSeq 2000 platform (Illumina, USA), employing 150 bp paired end reads. Quality control, assembly, annotation, and differential gene expression (DGE) analysis were performed from Novogene Europe. The reference genome used for mapping can be found under the GenBank ID GCA_900880675.1. For the detection of DGE, edgeR's exact test was used since there were no biological replicates [29–31]. DEGs were identified using a cutoff threshold of a log2FoldChange greater than 0.6 and a *p*-value less than 0.05. The detailed algorithm can be found in the public repository GitHub (https://github.com/RafaelAngelakopoulos/Bioz_lab/tree/main/RNAseq). The raw data from the transcriptome sequencing can be found under the project PRJNA1064006 in the SRA database.

### 2.9. Quantitative Expression Analysis (qRT-PCR)

Several DEGs from the RNAseq were selected to be further assessed through real-time quantitative PCR (qPCR). PCR was performed individually in the selected six families to determine the relative expression levels of certain DEGs. Primers were designed using the PrimerBlast tool (NCBI, https://www.ncbi.nlm.nih.gov/tools/primer-blast/) and evaluated for primer dimers hairpins formation, etc., with Beacon designer software (Premier Biosoft, http://www.premierbiosoft.com/qOligo/Oligo.jsp?PID=1, accessed on: 20/10/2020).

Serial dilutions of pooled samples were used to determine the efficiency of the reactions. A 10 μL reaction was performed using KAPA SYBR FAST qPCR Master Mix (2x) Kit (Kapa Biosystems, cat no: KK4618) with an appropriate concentration of each gene-specific primer set. The amplification steps were: 5 min at 95°C, followed by 40 cycles of 95°C for 20 s and 60°C for 20 s accordingly. A dissociation/melt curve step was added for the verification of the specificity of primers. Reactions were performed in duplicate and a maximum ±0.5 difference in Ct values between the duplicates was applied as a cutoff.

A series of genes were evaluated as reference genes for gene expression normalization using the RefFinder algorithm [32].

For the calculation of initial fluorescence the mathematical equation used was *R*_0_ = *T*/(*E* + 1)^Ct^, where *T* is a set threshold across genes, *E* is the PCR reaction efficiency for each gene, and Ct is the number of cycles to reach the set threshold [33]. Finally to normalize the relative gene expression of all genes the geometric mean of the three most stable genes was used as a normalization factor (*EF1a*, *RPL13*, and *RPS18*) [34]. The genes that were selected and the corresponding primers are presented in Table 2.

### 2.10. Statistical Analysis

Statistical analysis was conducted in RStudio, using the appropriate packages [35, 36]. The normality of the data was assessed using the Shapiro–Wilk test [37]. Data deviated from the normal distribution, hence nonparametric tests were selected for downstream analysis The Kruskal–Wallis test was employed to determine the influence of varied diets on the observed variables at a significance threshold of *p* ≤ 0.05 [38]. To illustrate the results, graphs were prepared using the R packages ggplot and ggpubr [39, 40].

Random forest was used as a classifier to reveal which candidate markers were mostly correlated with growth rate using the R package *randomForest* [41]. Random forest works using a “sampling with replacement approach.” A train dataset is used, leaving one-third of the data to run unbiased estimate of the classification error as trees are added to the forest estimating simultaneously the variable importance. After each tree is built, all the data are run down the tree and proximities are computed for each pair of cases [42]. The maximum number of trees was set at 9000. The model was then tuned using cross-validation performance, and its accuracy was evaluated using a different test dataset. Finally, the significance of each variable in the model was assessed using the model's mean decrease accuracy for each variable.

In parallel with random forest a multiple logistic regression was used using the *glm* R function. Multiple logistic regression finds the equation that best separates the two groups for the values of the *n*-variables classifying them according to their significance.

## 3. Results

### 3.1. Zootechnical Data

**Figure 1:**
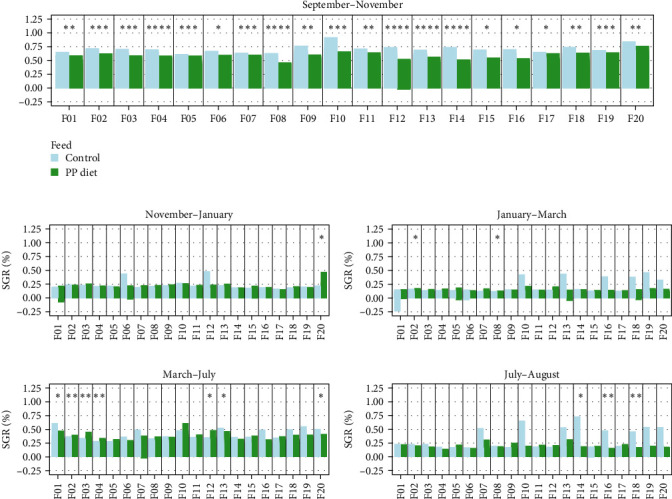
Comparison of periodical family SGR (median) between the two diets. (A) SGR September–November, (B) SGR November–January, (C) SGR January–March, (D) SGR March–July, and (E) SGR July–August. Light blue and green denote the families that are fed on the FM diet and the PP diet, respectively. Significance levels are presented on the plot (*⁣*^*∗*^*p* < 0.05, *⁣*^*∗∗*^*p* < 0.01, *⁣*^*∗∗∗*^*p* < 0.001, *⁣*^*∗∗∗∗*^*p* < 0.0001).

**Figure 2:**
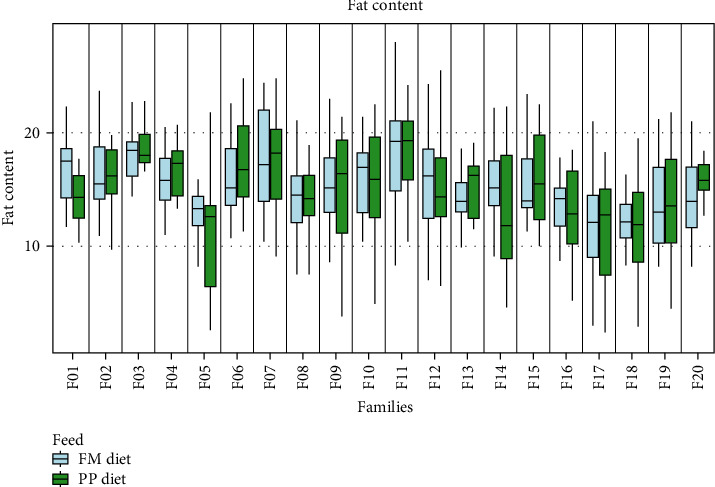
Muscle fat content measured at the end of the experiment. Light blue and green denote the families that are fed on the FM diet and the PP diet, respectively.

**Figure 3:**
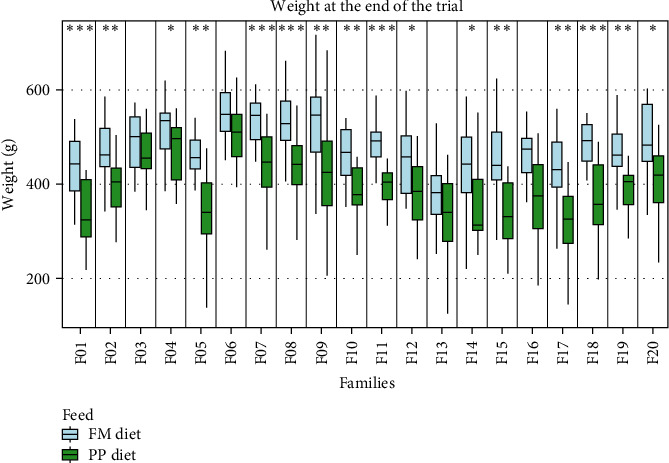
Body weight of all families at the end of the trial. Light blue and green denote the families that are fed on the FM diet and the PP diet, respectively. Significance levels are presented on the plot (*⁣*^*∗*^*p* < 0.05, *⁣*^*∗∗*^*p* < 0.01, *⁣*^*∗∗∗*^*p* < 0.001, and *⁣*^*∗∗∗∗*^*p* < 0.0001).

**Figure 4:**
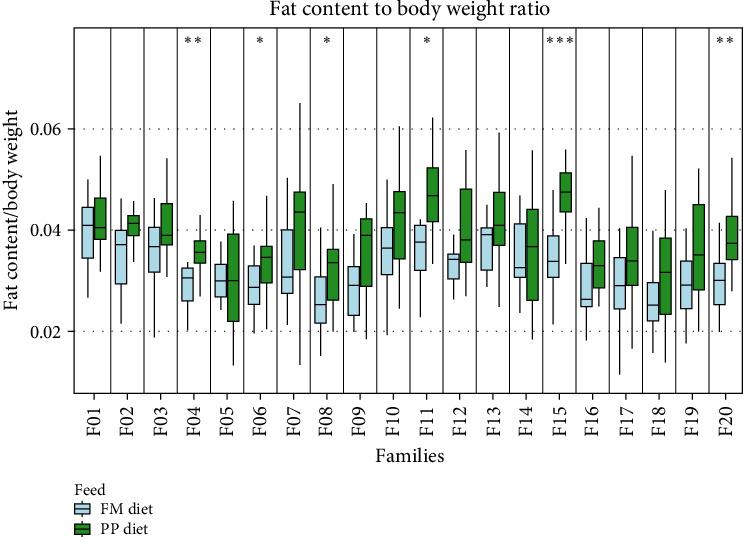
Fat content to body weight ratio of all families at the end of the trial. Light blue and green denote the families that are fed on the FM diet and the PP diet, respectively. Significance levels are presented on the plot (*⁣*^*∗*^*p* < 0.05, *⁣*^*∗∗*^*p* < 0.01, *⁣*^*∗∗∗*^*p* < 0.001, and *⁣*^*∗∗∗∗*^*p* < 0.0001).

Data on growth performance, for each sampling group, are shown in Figure 1 and Table S1. Statistically significant differences in SGR between the two dietary treatments were observed, that followed three different SGR patterns. Four families (F03, F04, F06, and F15) presented no differences in growth rate between the two dietary treatments; five families (F01, F08, F12, F14, and F20) exhibited different growth rates when fed on different feed during the first 4 months of the trial (September 2018–January 2019); finally, SGR was different in 11 families (F02, F05, F07, F09, F10, F11, F13, F16, F17, F18, and F19) fed on different diets only during September–November 2018 (Figure 1). Fat content was also measured after 1 year of rearing in sea cages with no statistically significant differences between the two feeding groups, although family-dependent differences were observed (Figure 2). Body weight measured at the end of the trial (August 2019) exhibited statistically significant differences in almost all families, with fish fed with the FM diet weighing more than fish fed on the PP diet (Figure 3). The fat content to body weight ratio was also assessed, with the PP diet group exhibiting a greater ratio (Figure 4).

### 3.2. Biochemical Markers

Growth and fattening of fish are two related processes that result in overall weight gain. To evaluate new nonlethal biochemical markers for the assessment of different feeds, triglyceride levels, cholesterol levels, and protein content of blood serum were assayed. Out of the 1000 individuals, 600 fish were retained for further analysis due to mortalities and/or failure to meet quality control criteria (e.g., hemolysis).

#### 3.2.1. Triglyceride Levels in Serum

**Figure 5:**
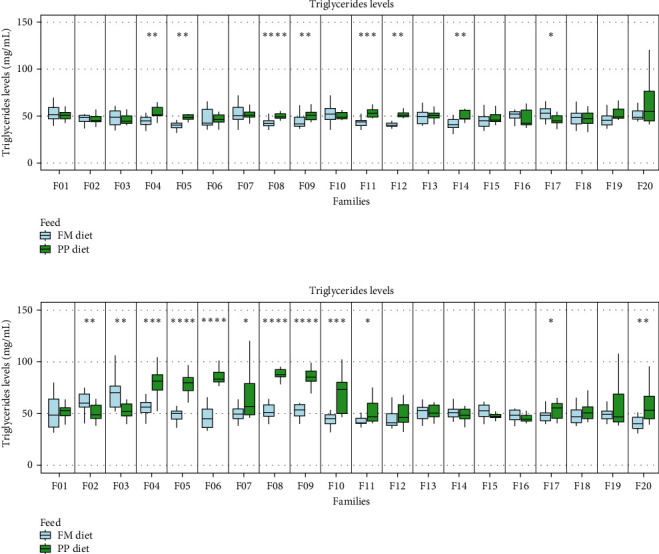
Triglycerides levels in blood serum (A) 15 days (D15) and (B) 30 days (D30) following the start of the feeding trial. Higher values were observed in the PP diet group 30 days after trial initiation. Significance levels are presented on the plot (*⁣*^*∗*^*p* < 0.05, *⁣*^*∗∗*^*p* < 0.01, *⁣*^*∗∗∗*^*p* < 0.001, and *⁣*^*∗∗∗∗*^*p* < 0.0001).

Higher statistically significant differences in triglyceride levels were observed on D30 of the trial, with higher values recorded in the PP diet group. Higher changes in triglyceride levels were observed on D30 in families F2–F11 compared to the mild changes observed on D15. Interestingly, families F12 and F14 showed significant differences in triglyceride levels on D15, but these differences seemed to equalize by D30 (Figure 5).

#### 3.2.2. Cholesterol Levels in Serum

**Figure 6:**
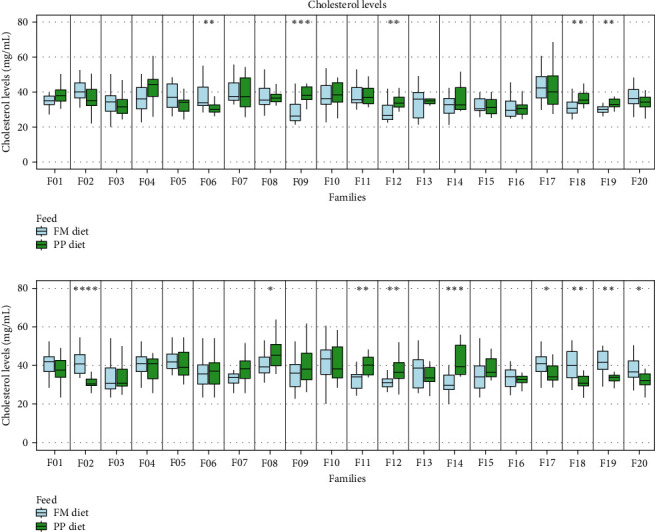
Cholesterol levels in blood serum (A) 15 days (D15) and (B) 30 days (D30) following the start of the feeding trial. Significance levels are presented on the plot (*⁣*^*∗*^*p* < 0.05, *⁣*^*∗∗*^*p* < 0.01, *⁣*^*∗∗∗*^*p* < 0.001, and *⁣*^*∗∗∗∗*^*p* < 0.0001).

Serum cholesterol was measured individually after 15 (D15) and 30 (D30) days of trial initiation (Figure 6). Comparable mean cholesterol values were recorded on both sampling days. Also, the overall median values among the families between the two dietary treatments were similar (PP diet [D15 : 35.15 mg/mL, D30 : 35.6 mg/mL]; FM diet [D15 : 34.14 mg/mL, D30 : 37.18 mg/mL]).

#### 3.2.3. Protein Content in Serum

**Figure 7:**
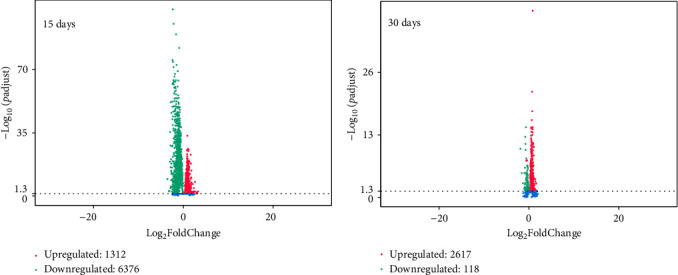
Volcano plots depicting genes upregulated in both dietary groups on the two sampling days: (A) D15 and (B) D30.

**Figure 8:**
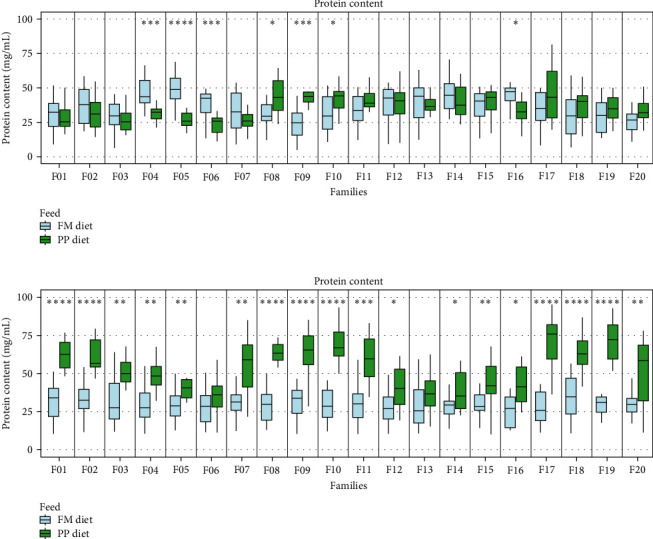
Protein content in blood serum (A) 15 days (D15) and (B) 30 days (D30) following the start of the feeding trial. Significance levels are presented on the plot (*⁣*^*∗*^*p* < 0.05, *⁣*^*∗∗*^*p* < 0.01, *⁣*^*∗∗∗*^*p* < 0.001, and *⁣*^*∗∗∗∗*^*p* < 0.0001).

Wide-ranging effects on serum protein content were observed on D30 in most families, with some families also showing an earlier response on D15, although this response was reversed on D30 (Families F04, F05, and F06). On D30, protein content was higher in all families within the PP diet group (Figure 7). The protein content of the FM diet group was similar on both sampling days (overall median D15 : 34.05 mg/mL, D30 : 29.73 mg/mL). For the PP diet group, higher protein values were observed on D30 compared with D15 (overall median D15 : 34.05 mg/mL D30 : 54.46 mg/mL) (Figure 8).

### 3.3. Transcriptome Analysis

#### 3.3.1. Differential Expression Analysis

Based on the previously identified SGR patterns (3.1), six families were selected for differential expression analysis. Two families that showed no differences in growth rate between the two dietary treatments (F06 and F15) were chosen. Additionally, two families that exhibited differences between the two dietary treatments in the time from September 2018 to January 2019 (F05 and F17), and two families with SGR differences between the two dietary treatments only from September to November 2018 (F08 and F20) were selected. Comparative transcriptomics analysis revealed that 6376 genes were upregulated in all families in the FM diet group compared with the PP diet group on D15. On D30, 2618 genes were upregulated in all families in the PP diet group (Figure 7).

**Figure 9:**
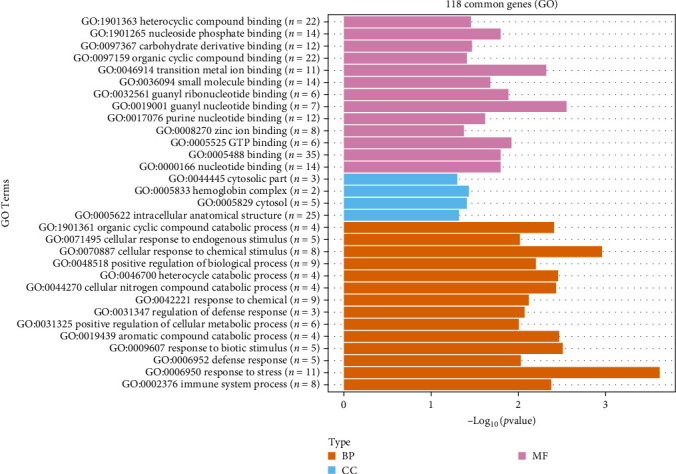
GO term enrichment analysis of the common DEGs in the three GO annotation domains: biological processes (BPs), cell components (CCs), and molecular functions (MFs).

Overall, 118 DEGs were common in all comparisons tested (Tables S2 and S3). A Gene Ontology annotation was performed using DAVID database (https://david.ncifcrf.gov/summary.jsp) to cluster the DEGs according to the biological processes their proteins are involved, their molecular function, and cellular localization (Figure 9).

Genes with the lowest *p*-value and the biggest logFoldChange associated with processes like stress response, oxidative phosphorylation, ubiquitin-mediated proteolysis, lipid metabolic processes, and immune system processes were selected, and primers were designed to assess them through real-time RT-PCR (Table 2).

#### 3.3.2. Family-Specific Responses

The detailed expression patterns revealed a significant family-specific response of erythrocyte transcriptome to the PP diet. In addition, the expression patterns differentiated between D15 and D30 after trial initiation, adding an extra dimension to the family-specific response (Table 3). Figures of the family-specific responses for each gene are provided in Figures S2–S17.

The expression of genes encoding for complement proteins (*c1ql3*, *c1ql4*) was increased in the families fed on PP diet, with *c1ql3* exhibiting the strongest and earliest response to the PP diet on D15, with elevated expression levels in all families (Table 3).

The PP diet led also to a higher cystatin expression in three families on D15 and four families (F06, F15, F17, and F20) on D30. The PP diet effect was consistent in families F15 and F17 on both sampling days (Table 3).

Three of the genes encoding for proteins of the oxidative phosphorylation complex (*ndufa2*, *rfe.sdt*, and *cox7a2l*) were significantly affected by the diet with the FF diet supporting higher expression levels; on D30 the expression of *ndufa2* and *cox7a2l* was significantly elevated in four (F08, F15, F17, and F20) and three (F08, F15, F17) families, respectively (Table 3).

#### 3.3.3. Marker Selection

A random forest approach combined with a logistic regression method was used to classify the set of biochemical and molecular variables affecting growth. Random forest was trained as a classification algorithm using the known quality, that is, high and low SGR. The variables were then further classified using a logistic regression model based on the influence of the two diets on SGR. In both cases, a growth prediction model was created and then utilized to categorize samples whose growth had been masked prior to analysis.

**Figure 10:**
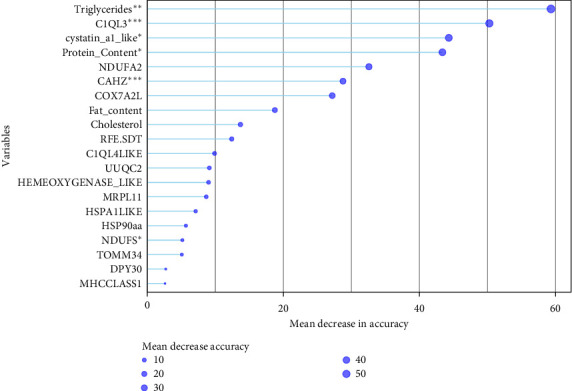
Random forest classifier. Graph shows the molecular and biochemical markers correlated with final growth in a hierarchical manner. Superscripts derive from the multiple regression analysis.

A combined classifier was produced using both models (Figure 10) with high accuracy (*A* > 80%) (Table 4). The classification of the variables was then interpreted as the gradient effect of growth on those variables, that is, the higher the variable impact on the model, the bigger their potential as markers. Variables qualified as potential markers are two biochemical markers (triglycerides and protein serum content) and five molecular markers (*C1QL3*, *cystatin_a1_like*, *NDUFA2*, *CAHZ*, and *COX7A2L*).

## 4. Discussion

It is evident from the literature that efforts have been made to enhance fish growth rates and adaptation to dietary changes with minimal impact on growth and health [18, 43–46]. The drawback of the existing procedure in aquaculture is that traits such as growth and adaptability are usually defined after harvest, leading to a long-distance journey [7]. Furthermore, until now, most markers developed have been tissue-based markers, requiring fish to be sacrificed for tissue collection adding to the problem. Following the recent trend in farming animals with increasing concern on welfare matters, peripheral blood is employed as an accessible and less invasive source of information that permits the examination of changes in different physiological states [47].

In this study, the two feeds were designed to be isoproteinic and isolipidic, with the FM diet group containing more FM and fish oils compared with the PP diet group which contained more plant-origin proteins and oils. PP sources, while less expensive and more readily available, may include antinutritional factors which can induce various physiological responses [10, 48].

### 4.1. Growth Performance

It is noteworthy that the fish fed with the PP diet exhibited a lower body weight compared with the FF diet group at the end of the trial (Figure 3); and as expected, the fat-to-body ratio was significantly higher in the PP diet group. This indicates that the weight gained by the fish fed on the PP diet was attributable to fat deposition. These findings are consistent with research, throughout the years, that shows how diets high in PP feeds stimulate lipid biosynthesis [49–53]. Furthermore, the feeding with the PP diet led to a lower SGR in the initial period (September–November), yet those differences tended to fade out during the winter period when water temperatures dropped (Figure S1) in conformity with growth rates. The differences in growth performance observed during the first 4 months are likely due to metabolic plasticity and adaptation to the diet composition. As the fish adapt to the new diet, their responses become more stable, leading to milder differences later in the trial. This suggests that the observed variations are more reflective of metabolic plasticity rather than differences in growth trajectories, especially given that the breeding program was designed to enhance growth rates over extended periods. Additionally, seasonal factors may also play a role in this adaptation process.

Water temperature is an important factor which influences growth rate of fish although temperature-growth relationships are not so clear, since this relationship is influenced by factors such as metabolic rate, feeding efficiency, and oxygen levels [54]. A drastic depression of metabolic rate and feed consumption is observed in gilthead sea bream when sea water temperature drops [55]. This so-called winter syndrome [56] among other factors such as decreased diet digestibility and depletion of fat deposits stagnates growth due to high energy cost [9, 57]. Considering the observed depression in metabolic rate within these families and the growth stall during the winter period, it is reasonable to suggest that replacing a percentage of FM with plant meal may not adversely affect fish growth. Although it should be noted that the possible effects on physiological responses such as stress, or the digestive tract of the fish induced by this replacement were not examined in the present study. The patterns of SGR were later reversed, coinciding with a rise in temperature (Figure 1). When exposed to cold, organisms increase the proportion of unsaturated fatty acids in the phospholipid fraction of their cell membranes to maintain proper function. In various species and tissues, adapting membranes to lower temperatures consistently results in higher levels of unsaturated fatty acids [58].

### 4.2. Biochemical Indices in Serum

Triglyceride levels were higher in most families fed with the PP diet on both sampling days (D15: 13/20 families, D30: 13/20 families). The differences were more pronounced on D30 compared to D15. Statistically significant differences were observed in several families (F02–F11, F17, and F20) on D30. These findings might be attributed to the fact that triglycerides are the most common type of dietary lipid present in fats and oils. As triacylglycerols are the main fat deposits in physiological states demanding the consumption of fuel reserves, fat mobilization is observed [58]. This is magnified under stressful conditions such as lower energy absorption where reserves are used. Since triglycerides are the primary fat deposit, the observed differences could indicate fat mobilization to compensate for lower energy supply. Cortisol inhibits glycogen synthesis and stimulates the mobilization of glucose and fatty acids in response to a stressful stimuli [59]. Lower growth rates and higher triglyceride content have been seen in cases of prolonged elevated cortisol levels [60].

Cholesterol levels revealed a mixed pattern. In many families, cholesterol levels were lower in the PP diet group. These findings are in accordance with a study where higher cholesterol levels in a diet richer in high-quality protein were observed [61]. Another explanation for lower levels of cholesterol in the PP diet group is that fish face difficulties exploiting plant lipids due to plants containing only a small amount of cholesterol and producing mainly phytosterols [62]. Additionally, genetic predisposition could be a contributing factor to the variability observed in the results, as different families may have varying responses to dietary lipid sources.

Finally, serum protein level was higher in most of the families the PP diet group. Since total proteins in serum are mostly synthesized in the liver, serum protein content may indirectly relate to liver function [63, 64]. It has been found that altering the diet of mostly carnivorous fish has resulted in liver hyperplasia or difficulties in adapting to the new feed, exploiting plant-derived proteins [65–69].

### 4.3. Family-Specific Responses to Diet

Over the last two decades, efforts have been made to improve the utilization of plant raw materials by fish, either by reducing the antinutritional factors and the subsequent negative effects on growth or via selection programs by improving genetically the “metabolic/physiological acceptability” of plant raw materials [16]. Fish used in the present study came from 20 full-sib families split into two feeding groups. Family-specific responses (Figures S2–S17) indicate the biological plasticity that it is possible to be manipulated through genetic selection, similar to other studies in gilthead sea bream [9, 17, 18], European sea bass [16], and rainbow trout [70].

### 4.4. Markers Selection

A marker selection analysis was performed under the assumption that whole blood mirrors the variations in metabolism. The data presented herein confirmed that the gene expression profiling in the blood represented a relevant source for identifying candidate biomarkers since a plant-rich diet (PP diet) triggered significant changes in specific biochemical (triglycerides and protein serum content) and molecular (*C1QL3*, *cystatin_a1_like*, *NDUFA2*, *CAHZ*, and *COX7A2L*) markers (Figure 10). Triglycerides, as mentioned, are a key form of stored energy, and their levels can reflect alterations in lipid metabolism, such as the mobilization of fat deposits due to dietary changes [53]. Similarly, protein serum content indicates overall protein metabolism and utilization. Changes in serum protein levels could suggest how efficiently these alternative proteins are being utilized for growth and maintenance.

The observed transcriptome changes likely reflect the fish's comprehensive physiological and metabolic response to the plant-rich diet, indicating how the fish are adapting. For example, changes in genes involved in protein turnover, such as cystatin_a1_like, might indicate an adaptation to better assimilate PPs, which may have lower digestibility and/or different protease inhibitory effects compared to FM.

Additionally, alterations in the expression of genes related to mitochondrial function and energy production (NDUFA2, COX7A2L) likely reflect how the fish are coping with the energy demands and nutrient availability associated with the plant-rich diet. Lastly, C1QL3 is a gene that suggests an immune or inflammatory response to the plant-rich diet, as plant ingredients can contain antinutritional factors or other compounds that may provoke a mild immune response. However, it should be noted that genes like C1QL3 can also participate in other physiological processes as interactive molecules. The observed changes in gene expression may be influenced by various, as-yet-unknown factors affecting growth. However, it is more likely that these changes reflect alterations in other biological functions that are linked with growth having an impact on overall physiology and behavior. While gene expression patterns can serve as valuable indicators, they are more likely to signify broader physiological shifts rather than directly driving growth.

In the current study, it was possible to discriminate between the two groups with high accuracy, with 87% and 83% when using the random forest and multiple regression approaches, respectively, using these three biochemical and five molecular markers. Similarly, other studies showed that blood is a good and easy-to-obtain material that can be a good source of information regarding growth [25, 27]. Thus, blood can be a good source of information regarding physiological changes from alternative feeds in gilthead seabream.

## 5. Conclusion

This study contributed to a better understanding of mechanisms and pathways that are activated upon FM replacement by plant-derived proteins as early as 30 days after the introduction of a plant-based diet (PP diet). Hence, it can be the first step in the selection of early biochemical and molecular biomarkers useful for the development of new alternative feeds in gilthead seabream farming, taking into consideration the family-specific responses that were recorded. This highlights a useful plasticity that can be manipulated through genetic selection breeding programs. Additional studies are required to confirm the caliber of the predictors on different lines of fish before these biomarkers can be commercially used.

## Figures and Tables

**Table 1:** Formulation and chemical composition of feeds expressed as percentage (%) of dry weight.

Ingredients (%)	FM diet	PP diet
Fish meal standard	24.6	—
Fish meal LT	—	8.1
Fish oil	4.6	5.0
Salmon oil	6.5	—
Rapeseed oil	—	6.5
Soya bean meal	9.9	21.0
Rapeseed meal	—	14.0
Soya protein concentrate	17.0	7.0
Corn gluten	14.5	9.6
Plant premix^a^	6.6	8.0
Sunflower meal	5.0	4.1
Wheat	9.6	10.7
Amino acid premix^b^	—	3.2
Vitamin and mineral premix	1.0	1.0
Phospholipids^c^	0.3	0.8
Ca and P source^d^	0.4	1.0

Proximate analysis	FM diet	PP diet

Protein	47.8	42.7
Lipids	18.3	17.5
Ash	6.8	5.9
Fiber	2.9	4.5
Humidity	7.6	8.7
Fatty acids profile (%)		
C14 : 0	0.56	0.32
C16 : 0	1.67	1.04
C18 : 0	0.45	0.31
C18 : 1	5.27	5.51
C18 : 2	1.82	1.85
C18 : 3	0.95	0.93
Total *ω*−3	3.26	2.46
Total *ω*−6	2.18	2.11
Total *ω*−9	6.35	5.68
Unsaturated	13.28	11.22
Saturated	3.18	2.26
MUFA	7.79	7.14
PUFA	5.45	4.01
C22 : 6 DHA	1.03	0.76
C20 : 5 EPA	0.81	0.63
C20 : 4 ARA	0.06	0.06
EPA/DHA	0.78	0.83
EPA/ARA	14.05	11.69
DHA/ARA	18.00	14.16

^a^Mix of single-cell protein, by-products of amino-acids production, and by-products of nGMO cereals' fermentation.

^b^L-Lysine 79%, DL-methionine 99%, L-threonine 98.5%.

^c^Hydrolyzed lecithin source FRA lecimax.

^d^Monocalcium phosphate and carbon carbonate.

**Table 2:** Genes selected for validation of differential expression through real-time RT-PCR.

Gene ID	Gene description	Gene name	Forward primer	Reverse primer	Product size (bp)
ENSSAUG00010018560/XM_030411990.1	Elongation factor 1-alpha, somatic form	*ef1a*	TCAAGGGATGGAAGGTTGAG	AGTTCCAATACCGCCGAT	152
ENSSAUG00010003114	Ribosomal protein L13a	*rpl13*	TCTGGAGGACTGTCAGGGGCATGC	AGACGCACAATCTTAAGAGCAG	197
ENSSAUG00010000811	40S ribosomal protein S18	*rps18*	AGGGTGTTGGCAGACGTTAC	GAGGACCTGGCTGTATTTGC	148
ENSSAUG00010012992	Complement C1q-like protein 3	*c1ql3*	TTTGGAGACGGAGCGAAGAC	CCATACGCCTCACCACCTTT	121
ENSSAUG00010012990	Complement C1q-like protein 4	*c1ql4*	AGGTTGACACAGCCTTCCATA	CACTCATGTTGGGTTTGCAGG	111
ENSSAUG00010011851	Carbonic anhydrase	*cahz*	AGGTGGACTTTGTGGACGAC	AAGCTCACAGGGGAACTTGA	155
ENSSAUG00010002150	NADH:ubiquinone oxidoreductase subunit A2	*ndufa2*	CAGTAAGGGGGCCAGAGATT	GTTGTCCACCATGACACTGC	157
ENSSAUG00010023048	Cytochrome c oxidase subunit 7A-related protein, mitochondrial-like	*cox7a2l*	CGGTGTGTTGTGAGCAGAAA	CGTCACTCCGTTCAGCTTGT	100
ENSSAUG00010003856	Rieske (Fe-S) domain containing	*rfe.sdt*	AGATGTGCATCGTTTGTCCA	TAGACATCCCCGTTGGTCTC	166
ENSSAUG00010006080	NADH:ubiquinone oxidoreductase core subunit S1	*ndufs*	CCCACTCTTCAACGCCAGAA	TCCCAGGTGGTCATACGAGT	106
ENSSAUG00010021563	Ubiquinol-cytochrome-c reductase complex assembly factor 2	*uuqc2*	TGAAGCTGTGTGAGGAATGG	CGGGCCAAACTTTCATACAT	157
ENSSAUG00010014574/LOC115570099	Cystatin-A1-like	*cystatin*	GAGTGAGACAAGGGATGCTGA	TGAATGTAGCTGGGTCCTCC	180
ENSSAUG00010006606	Mitochondrial ribosomal protein L11	*mrpl11*	ACGAGATCGCAAGGGTTAAA	GCTGCTCCAGGAAGATTTTG	159
ENSSAUG00010007642	Translocase of outer mitochondrial membrane 34	*tomm34*	CCTGTCGGTGAAGCAGTACA	AGGTTGTTCAGGTCGTCCAC	150
ENSSAUG00010025859	Heme oxygenase-like	*hmo2*	CGCCTACACCCGTTATCTGG	GCTGTTCATCCTGCTCCTGT	166
ENSSAUG00010012636/LOC115576233	Heat shock protein family A	*hspa1l*	GTACGGTCTGGACAAAGGCA	GTCAAAGTCTTCTCCGCCCA	157
ENSSAUG00010025665	Heat shock protein HSP 90-alpha	*hsp90aa*	TGACCCTCAGACACACTCCA	GTCGTCATCGTCCCCTTCAA	139
ENSSAUG00010000727	Major histocompatibility complex class I-related gene protein-like	*mhc class 1*	AGATCGGATCGGAACCAACG	CGATGAATCCAACAGCACCG	107
ENSSAUG00010011097	dpy-30 histone methyltransferase complex regulatory subunit	*dpy30*	GTGCTCGCCAAGGACAGAC	TCTCCTCAAACTGGGATTTGTTCT	82

**Table 3:** Statistically significant differences per family, D15 and D30 after trial initiation.

Sampling day	D15						D30					
Gene/families	F05	F06	F08	F15	F17	F20	F05	F06	F08	F15	F17	F20
*c1ql4*	—	⁣∗∗	—	—	⁣∗	—	*—*	*—*	—	*—*	*—*	⁣∗
*c1ql3*	⁣∗∗	⁣∗	⁣∗∗	⁣∗∗	⁣∗	⁣∗	*—*	⁣∗∗	—	*—*	*—*	*—*
*cahz*	—	⁣∗∗	⁣∗	—	—	—	*—*	*—*	⁣∗∗∗	*—*	*—*	⁣∗
*ndufa2*	*⁣* ^*∗∗*^	—	—	—	—	—	*—*	*—*	*⁣* ^*∗∗∗*^	*⁣* ^*∗∗*^	*⁣* ^*∗∗*^	*⁣* ^*∗∗∗*^
*cox7a2l*	—	*⁣* ^**∗** **∗** **∗**^	—	—	—	—	*—*	*—*	*⁣* ^*∗∗*^	*⁣* ^*∗∗∗*^	*⁣* ^*∗∗*^	*—*
*rfe.sdt*	—	—	*⁣* ^**∗** **∗** **∗**^	*⁣* ^**∗** **∗**^	—	—	*—*	*⁣* ^*∗∗*^	*—*	*—*	*—*	*—*
*ndufs*	—	—	⁣∗∗∗	*⁣* ^**∗**^	—	—	*—*	*—*	*—*	*—*	*—*	⁣∗
*uuqc2*	—	⁣∗	—	—	—	—	*—*	⁣∗	*—*	*—*	⁣∗	*—*
*cystatin*	—	—	⁣∗∗	⁣∗	—	⁣∗	*—*	⁣∗	*—*	⁣∗∗∗	⁣∗	⁣∗∗∗
*mrpl11*	—	—	—	—	—	—	⁣∗∗	*—*	*—*	*—*	*—*	*—*
*tomm34*	*⁣* ^**∗** **∗**^	—	—	—	—	⁣∗	*—*	*—*	*—*	*—*	*—*	*—*
*hmo2*	—	⁣∗	—	—	*⁣* ^**∗**^	—	*—*	*—*	*—*	*—*	*—*	⁣∗∗∗
*hspa1l*	—	—	*⁣* ^**∗**^	—	—	—	*—*	*—*	*—*	*—*	*—*	⁣∗∗∗
*hsp90aa*	⁣∗∗	—	*⁣* ^**∗**^	—	⁣∗	—	*—*	*⁣* ^*∗∗*^	*—*	*—*	*—*	*—*
*mhc class 1*	*⁣* ^**∗**^	—	—	—	—	—	*—*	*—*	*—*	*—*	⁣∗	⁣∗∗∗
*dpy30*	—	⁣∗	—	—	—	⁣∗	⁣∗	*⁣* ^*∗*^	*⁣* ^*∗∗*^	*⁣* ^*∗*^	*—*	*—*

*Note:* Asterisks denote statistically significant differences between feeds per family and sampling day. Genes higher expression for the FM diet are marked bold when for the PP diet are underlined (*⁣*^*∗*^*p* < 0.05, *⁣*^*∗∗*^*p* < 0.01, *⁣*^*∗∗∗*^*p* < 0.001, and *⁣*^*∗∗∗*^*p* < 0.0001).

**Table 4:** Accuracy, sensitivity, and specificity of random forest and multiple regression algorithm.

Model	Accuracy	Sensitivity	Specificity	Evaluation metric
Random forest	0.87	0.91	0.83	SGR
Multiple regression	0.83	—	—	

## Data Availability

Transcriptome sequencing data used in this study are available through SRA (BioProject ID PRJNA1064006).

## Supporting Information

Supporting Information The file contains: Table S1. Zootechnical data throughout the experiment per family; Table S2. Table of genes from RNAseq for each family; Table S3. List of common genes between comparisons; Figure S1. Sea temperature and oxygen variation throughout the feeding trial; Figures S2–S17. Gene expression of the markers in the erythrocytes per family.
