# Supplementary material for: Selection of Nonlethal Early Biomarkers to Predict Gilthead Seabream (Sparus aurata) Growth
Source: Aquac Nutr. 2025 Jan 6;2025:9918595. doi: 10.1155/anu/9918595 (PMC11730015; doi:10.1155/anu/9918595)
Supplement: Supporting Information — The file contains: Table S1. Zootechnical data throughout the experiment per family; Table S2. Table of genes from RNAseq for each family; Table S3. List of common genes between comparisons; Figure S1. Sea temperature and oxygen variation throughout the feeding trial; Figures S2–S17. Gene expression of the markers in the erythrocytes per family. [file 9918595.f1.docx]

**Supplementary Table 1.** Zootechnical data throughout the experiment per family (Control diet/Trial diet). IBW (Initial body weight), FBW (Final body weight), SGR1 (SGR: September-November), SGR2 (SGR: November-January), SGR3 (SGR: January-March), SGR4 (SGR: March-July), SGR5 (SGR: July-August), Final SGR (SGR: September-August)

| Families | IBW (g) | FBW (g) | SGR1 (%) | SGR2 (%) | SGR3 (%) | SGR4 (%) | SGR5 (%) | Final SGR (%) |
| --- | --- | --- | --- | --- | --- | --- | --- | --- |
| F01 | 42/37 | 438/338 | 0.56/0.46 | 0.15/0.13 | 0.09/0.1 | 0.28/0.32 | 0.17/0.16 | 1.89/1.76 |
| F02 | 41/37 | 466/385 | 0.6/0.46 | 0.15/0.14 | 0.1/0.14 | 0.29/0.35 | 0.17/0.16 | 1.96/1.83 |
| F03 | 43/44 | 490/464 | 0.59/0.49 | 0.17/0.17 | 0.1/0.11 | 0.29/0.34 | 0.15/0.15 | 1.93/1.86 |
| F04 | 47/47 | 521/472 | 0.62/0.52 | 0.17/0.18 | 0.12/0.13 | 0.25/0.28 | 0.13/0.12 | 1.87/1.81 |
| F05 | 50/46 | 449/343 | 0.54/0.46 | 0.14/0.14 | 0.11/0.09 | 0.24/0.21 | 0.14/0.15 | 1.77/1.58 |
| F06 | 56/54 | 556/512 | 0.55/0.51 | 0.17/0.18 | 0.1/0.11 | 0.27/0.27 | 0.13/0.12 | 1.85/1.81 |
| F07 | 49/45 | 536/421 | 0.58/0.47 | 0.17/0.17 | 0.11/0.12 | 0.26/0.27 | 0.15/0.16 | 1.88/1.73 |
| F08 | 54/51 | 533/436 | 0.53/0.4 | 0.16/0.16 | 0.1/0.12 | 0.29/0.31 | 0.14/0.15 | 1.79/1.66 |
| F09 | 45/40 | 530/421 | 0.61/0.52 | 0.18/0.18 | 0.12/0.11 | 0.27/0.28 | 0.15/0.15 | 2/1.87 |
| F10 | 29/26 | 454/373 | 0.67/0.56 | 0.19/0.19 | 0.15/0.14 | 0.34/0.38 | 0.19/0.16 | 2.11/2.05 |
| F11 | 42/38 | 489/395 | 0.6/0.5 | 0.15/0.17 | 0.11/0.1 | 0.3/0.33 | 0.15/0.15 | 1.95/1.83 |
| F12 | 39/42 | 450/374 | 0.59/0.41 | 0.17/0.16 | 0.12/0.14 | 0.28/0.32 | 0.14/0.15 | 1.95/1.73 |
| F13 | 35/33 | 377/335 | 0.58/0.46 | 0.17/0.16 | 0.12/0.08 | 0.25/0.32 | 0.18/0.19 | 1.8/1.77 |
| F14 | 41/47 | 434/360 | 0.57/0.43 | 0.14/0.13 | 0.11/0.1 | 0.28/0.27 | 0.2/0.15 | 1.75/1.6 |
| F15 | 53/49 | 439/343 | 0.51/0.44 | 0.15/0.12 | 0.1/0.08 | 0.22/0.24 | 0.14/0.14 | 1.75/1.62 |
| F16 | 43/45 | 474/372 | 0.56/0.46 | 0.17/0.15 | 0.15/0.11 | 0.3/0.26 | 0.22/0.13 | 1.83/1.72 |
| F17 | 52/43 | 423/320 | 0.52/0.46 | 0.14/0.12 | 0.08/0.08 | 0.23/0.24 | 0.14/0.15 | 1.73/1.6 |
| F18 | 44/41 | 486/368 | 0.59/0.49 | 0.16/0.17 | 0.13/0.1 | 0.3/0.27 | 0.16/0.12 | 1.9/1.75 |
| F19 | 35/35 | 464/375 | 0.63/0.51 | 0.17/0.14 | 0.14/0.11 | 0.31/0.33 | 0.19/0.16 | 1.98/1.88 |
| F20 | 37/34 | 479/407 | 0.63/0.52 | 0.17/0.22 | 0.13/0.11 | 0.3/0.35 | 0.19/0.15 | 2.03/1.99 |

**Supplementary Table 2.** Table of genes with logFC and pvalue for each family. A15: experimental feed fifteen days after diet differentiation; A30: experimental feed thirty days after diet differentiation; B15: control feed fifteen days after diet differentiation; B30: control feed thirty days after diet differentiation

| F05A15vsF05B15 | | | |  | | F05A30vsF05B30 | | | |  | | F05A15vsF05A30 | | | |  | | F05B15vsF05B30 | | |
| --- | --- | --- | --- | --- | --- | --- | --- | --- | --- | --- | --- | --- | --- | --- | --- | --- | --- | --- | --- | --- |
|  | logFC | PValue |  | |  | | logFC | PValue |  | |  | | logFC | PValue |  | |  | | logFC | PValue |
| C1QL4(like) | -4.33 | 9.02E-68 |  | | CDA | | -2.32 | 1.71E-24 |  | | HBA1-like | | -4.92 | 3.41E-90 |  | | HSPA1L | | -4.81 | 4.15E-89 |
| C1QL4 like | -3.73 | 1.09E-57 |  | | plk1 | | -3.68 | 3.69E-23 |  | | HSPA1-like | | -4.52 | 3.05E-81 |  | | HSPA1-like | | -4.74 | 2.09E-87 |
| lncRNA | -4.73 | 3.02E-57 |  | | lncRNA | | -2.27 | 1.81E-22 |  | | HSPA1L | | -4.26 | 6.46E-74 |  | | DNAJB1 | | -4.23 | 5.15E-68 |
| gadd45g | -3.72 | 1.04E-55 |  | | ZCCHC17 | | -3.23 | 4.03E-22 |  | | HBA1 like | | -3.48 | 1.22E-53 |  | | Hr38 | | -3.62 | 1.33E-54 |
| LOC115574304 | -3.94 | 2.42E-51 |  | | LOC115574304 | | -2.09 | 1.85E-21 |  | | nr4a3 | | -4.25 | 1.12E-46 |  | | C1QL4-like | | 3.99 | 8.26E-50 |
| C1QL4-like | -3.84 | 2.62E-45 |  | | gdf15 | | -3.41 | 2.75E-19 |  | | Hr38 | | -3.46 | 7.06E-46 |  | | HSP90AA1 | | -3.25 | 5.70E-48 |
| CDA | -3.96 | 3.09E-41 |  | | nr4a3 | | 2.62 | 2.12E-17 |  | | DNAJB1 | | -2.88 | 2.21E-35 |  | | HBA1-like | | -3.02 | 9.57E-42 |
| LOC115593915 | -2.64 | 6.12E-34 |  | | meiob | | -2.15 | 5.56E-17 |  | | HBA1 | | -2.82 | 7.63E-34 |  | | heme oxygenase like | | -4.15 | 2.02E-38 |
| cystatin-A1L | -2.63 | 7.94E-34 |  | | ccnb1 | | -3.40 | 2.04E-16 |  | | JUNB | | -2.60 | 6.31E-33 |  | | nr4a3 | | -2.79 | 4.05E-32 |
| LOC115593917 | -2.64 | 1.32E-33 |  | | cystatin-A1 like | | -2.36 | 2.11E-16 |  | | HSP90AA1 | | -2.44 | 2.02E-29 |  | | COX7A2L | | -3.07 | 7.09E-31 |
|  |  |  |  | |  | |  |  |  | |  | |  |  |  | |  | |  |  |
| F06A15vsF06B15 | | | |  | | F06A30vsF06B30 | | | |  | | F06A15vsF06A30 | | | |  | | F06B15vsF06B30 | | |
|  | logFC | PValue |  | |  | | logFC | PValue |  | |  | | logFC | PValue |  | |  | | logFC | PValue |
| dpy-30 | -5.40 | 4.98E-35 |  | | IFI44-like | | 10.96 | 4.58E-43 |  | | HBA1-like | | -4.57 | 2.29E-80 |  | | Hr38 | | -4.22 | 2.62E-67 |
| Havcr2 | 2.42 | 1.13E-28 |  | | cystatin-A1 like | | 3.62 | 6.48E-42 |  | | HBA1 like | | -3.92 | 1.42E-63 |  | | HSPA1-like | | -3.66 | 3.34E-58 |
| IFI44 | 6.43 | 1.14E-22 |  | | C1QL4(like) | | 3.41 | 4.18E-37 |  | | HSPA1-like | | -2.99 | 1.09E-41 |  | | HSPA1L | | -3.49 | 1.33E-53 |
| C1QL4 | 5.15 | 3.87E-22 |  | | IFI44 | | -7.20 | 1.64E-31 |  | | HSPA1L | | -3.00 | 1.14E-41 |  | | nr4a3 | | -3.66 | 1.08E-47 |
| MHC-class1-like | 4.79 | 4.51E-22 |  | | dpy-30 | | 3.99 | 4.40E-31 |  | | LOC115583774 | | -3.15 | 3.03E-31 |  | | MX1 | | 3.31 | 1.70E-47 |
| Hr38 | 2.05 | 3.29E-21 |  | | COX7A2L | | -3.01 | 1.77E-27 |  | | CREM | | -2.48 | 1.80E-30 |  | | HBA1-like | | -3.27 | 1.21E-46 |
| MYEF2-like | -2.35 | 2.65E-20 |  | | MYEF2L | | 3.05 | 1.90E-22 |  | | JUNB | | -2.37 | 4.19E-28 |  | | heme oxygenase like | | -4.76 | 1.43E-43 |
| KDM4A-like | 2.74 | 3.18E-20 |  | | Havcr2 | | 2.04 | 1.45E-21 |  | | IFI44 | | 6.86 | 6.25E-28 |  | | dpy-30 | | 5.76 | 1.93E-42 |
| COX7A2L | -2.31 | 5.37E-20 |  | | ATRX-like | | 2.61 | 3.49E-21 |  | | MHC-class1 like | | 2.97 | 2.15E-27 |  | | RTP3-like | | 3.01 | 6.15E-42 |
| nr4a3 | 2.09 | 6.02E-20 |  | | NEU4-like | | 2.03 | 1.40E-15 |  | | gtpbp1 | | 2.33 | 4.22E-27 |  | | Heme oxygenase-like | | -4.13 | 2.65E-33 |

| F08A15vsF08B15 | | |  | F08A30vsF08B30 | | |  | F08A15vsF08A30 | | |  | F08B15vsF08B30 | | |
| --- | --- | --- | --- | --- | --- | --- | --- | --- | --- | --- | --- | --- | --- | --- |
|  | logFC | PValue |  |  | logFC | PValue |  |  | logFC | PValue |  |  | logFC | PValue |
| C1QL4 like | -4.01 | 3.96E-53 |  | C1QL4-like | -4.80 | 2.81E-73 |  | HBA1-like | -4.70 | 2.60E-86 |  | HSPA1L | -4.72 | 1.25E-86 |
| LOC115594449 | 4.39 | 1.65E-43 |  | MHC-class1L | -4.61 | 5.64E-48 |  | HBA1 like | -4.17 | 8.07E-72 |  | HSPA1-like | -4.63 | 2.01E-84 |
| gadd45g | -3.10 | 4.52E-40 |  | cystatin-A1 like | 2.94 | 1.13E-34 |  | C1QL4-like | 4.05 | 8.57E-56 |  | DNAJB1 | -4.05 | 1.83E-63 |
| dpy-30 | -5.51 | 8.09E-38 |  | dpy-30 | -3.48 | 6.51E-34 |  | HSPA1L | -3.44 | 2.05E-52 |  | Hr38 | -3.93 | 2.15E-62 |
| KAT7-like | 2.98 | 1.50E-37 |  | apoptosis-associated speck-like | -2.38 | 3.43E-27 |  | MHC-class1L | 6.49 | 2.18E-51 |  | cystatin-A1 like | 4.62 | 2.58E-59 |
| SAMD9L | 2.74 | 7.80E-34 |  | CDA | -2.05 | 2.31E-18 |  | HSPA1-like | -3.34 | 2.44E-50 |  | nr4a3 | -3.44 | 6.04E-46 |
| hykk | 3.70 | 3.29E-33 |  | COX8A | -2.15 | 2.76E-16 |  | JUNB | -2.71 | 1.67E-35 |  | heme oxygenase like | -4.05 | 1.17E-40 |
| Hr38 | 2.62 | 1.06E-32 |  | rgs7 | -2.04 | 1.46E-15 |  | HBA1 | -2.74 | 2.26E-34 |  | HBA1-like | -2.89 | 3.49E-39 |
| LOC115586187 | 3.07 | 1.75E-31 |  | C1QL4L | -2.54 | 2.60E-15 |  | heme oxygenase like | -3.67 | 1.95E-30 |  | HSP90AA1 | -2.54 | 1.06E-31 |
| HIPK3 | 2.62 | 9.77E-30 |  | C1QL3-like | -1.60 | 2.45E-14 |  | Hbb-b2 | -2.62 | 1.92E-28 |  | HBA1 like | -2.50 | 1.12E-30 |
|  |  |  |  |  |  |  |  |  |  |  |  |  |  |  |
| F15A15vsF15B15 | | |  | F15A30vsF15B30 | | |  | F15A15vsF15A30 | | |  | F15B15vsF15B30 | | |
|  | logFC | PValue |  |  | logFC | PValue |  |  | logFC | PValue |  |  | logFC | PValue |
| dpy-30 | -5.63 | 9.31E-44 |  | LOC115590011 | -3.30 | 1.21E-30 |  | HSPA1-like | -4.60 | 9.28E-84 |  | HSPA1L | -4.70 | 2.15E-86 |
| PGBD3 | 3.08 | 7.28E-36 |  | COX7A2L | 2.99 | 2.57E-30 |  | HSPA1L | -4.56 | 1.22E-82 |  | HSPA1-like | -4.67 | 2.10E-85 |
| NLRC3-like | 2.56 | 3.23E-29 |  | dpy-30 | 2.22 | 4.35E-21 |  | HBA1-like | -4.46 | 1.72E-79 |  | DNAJB1 | -4.21 | 1.27E-68 |
| C1QL3-like | 2.35 | 1.41E-27 |  | LOC115593923 | -2.41 | 9.71E-21 |  | HBA1 like | -3.56 | 1.10E-55 |  | dpy-30 | 6.54 | 1.69E-65 |
| LOC115593923 | 4.86 | 2.13E-25 |  | LOC115575516 | 4.06 | 9.76E-19 |  | asteroid homolog1-like | 3.40 | 1.53E-48 |  | MHC-class1L | 5.64 | 7.56E-60 |
| C1QL4-like | 3.18 | 2.11E-23 |  | LOC115583774 | 2.50 | 7.39E-16 |  | LOC115593923 | 6.05 | 2.35E-48 |  | heme oxygenase like | -4.92 | 4.61E-59 |
| NLRC3L | 2.41 | 3.26E-23 |  | TRAV25 | 2.17 | 1.12E-15 |  | MHC-class1L | 4.95 | 2.40E-46 |  | *HSP90AA1* | -3.21 | 6.47E-47 |
| KMT2C | 2.12 | 1.11E-22 |  | MBTPS2 | 4.98 | 2.49E-13 |  | RNF14-like | 3.68 | 2.46E-41 |  | Hr38 | -3.07 | 4.53E-42 |
| KAT7-like | 2.15 | 2.67E-22 |  | CREM | 1.52 | 3.00E-13 |  | heme oxygenase like | -4.09 | 8.70E-41 |  | HBA1-like | -2.92 | 9.78E-40 |
| MOG-like | 2.56 | 1.20E-21 |  | C1QL4 | 1.83 | 4.00E-13 |  | HSP90AA1 | -2.91 | 6.83E-40 |  | HSP30-like | -6.18 | 6.43E-37 |

| F17A15vsF17B15 | | |  | F17A30vsF17B30 | | |  | F17A15vsF17A30 | | |  | F17B15vsF17B30 | | |
| --- | --- | --- | --- | --- | --- | --- | --- | --- | --- | --- | --- | --- | --- | --- |
|  | logFC | PValue |  |  | logFC | PValue |  |  | logFC | PValue |  |  | logFC | PValue |
| SAA | -5.45 | 7.52E-50 |  | MYEF2-like | 2.68 | 2.79E-26 |  | HBA1-like | -4.35 | 1.07E-76 |  | HSPA1-like | -4.88 | 1.90E-91 |
| dpy-30 | -4.95 | 1.81E-42 |  | nuclear factor 7 brain-like | -3.61 | 1.50E-25 |  | HBA1 like | -3.95 | 5.91E-66 |  | HSPA1L | -4.81 | 2.04E-89 |
| LOC115574304 | -4.75 | 7.44E-35 |  | Ig lambda-1 light chain | -3.37 | 1.94E-22 |  | HSPA1-like | -3.40 | 9.84E-52 |  | heme oxygenase like | -4.98 | 5.72E-52 |
| Mannose-specific lectin | -4.57 | 8.56E-34 |  | GTPase 5 | 2.36 | 3.29E-19 |  | HSPA1L | -3.38 | 3.50E-51 |  | DNAJB1 | -3.52 | 4.71E-50 |
| KMT2C | 2.64 | 3.19E-33 |  | SAMHD1 | 2.95 | 3.23E-17 |  | heme oxygenase like | -4.54 | 2.02E-47 |  | Heme oxygenase-like | -4.56 | 2.53E-45 |
| samd3 | 2.60 | 3.12E-30 |  | coagulation factorVIII-like | 1.74 | 3.03E-16 |  | Hr38 | -3.45 | 2.78E-47 |  | Hr38 | -3.26 | 6.29E-45 |
| NLRC3-like | 2.42 | 4.19E-27 |  | wnk4 | 1.71 | 5.33E-16 |  | Mannose-specific lectin | -6.24 | 2.13E-43 |  | HBA1-like | -2.97 | 3.80E-41 |
| NLRC3L | 2.58 | 5.53E-27 |  | Ahsa1-like | 2.70 | 6.14E-14 |  | SAA | -4.29 | 1.99E-41 |  | HSP90AA1 | -2.75 | 2.30E-36 |
| hepcidin-like | -2.40 | 1.61E-26 |  | CREM | 1.56 | 8.96E-14 |  | HBA1 | -2.96 | 2.97E-39 |  | HBA1 like | -2.62 | 3.12E-33 |
| lncRNA | -4.71 | 1.98E-26 |  | SLC6A8-like | 1.79 | 1.16E-13 |  | dpy-30 | -4.20 | 3.83E-38 |  | C1QL3-like | 2.57 | 2.69E-32 |
|  |  |  |  |  |  |  |  |  |  |  |  |  |  |  |
| F20A15vsF20B15 | | |  | F20A30vsF20B30 | | |  | F20A15vsF20A30 | | |  | F20B15vsF20B30 | | |
|  | logFC | PValue |  |  | logFC | PValue |  |  | logFC | PValue |  |  | logFC | PValue |
| COX7A2L | -4.04 | 7.02E-48 |  | C1QL4 | 3.97 | 3.41E-53 |  | HSPA1L | -5.08 | 6.24E-97 |  | HSPA1-like | -4.64 | 1.55E-84 |
| KMT2C | 3.23 | 1.02E-46 |  | MHC-class1L | -4.95 | 1.05E-32 |  | HBA1-like | -4.99 | 1.16E-94 |  | HSPA1L | -4.53 | 3.01E-81 |
| LOC115578682 | -3.20 | 1.68E-43 |  | dpy-30 | 4.01 | 6.16E-30 |  | HSPA1-like | -4.93 | 8.25E-93 |  | C1QL4 | 5.14 | 8.26E-70 |
| C1QL4(like) | -2.77 | 2.01E-32 |  | COX7A2L | -3.13 | 2.78E-29 |  | HBA1 like | -4.62 | 4.13E-84 |  | HBA1-like | -3.52 | 6.88E-55 |
| HIPK3 | 2.66 | 6.02E-31 |  | C1QL4.like | -2.59 | 1.99E-21 |  | DNASE1L1 | -4.69 | 2.93E-63 |  | Hr38 | -3.37 | 4.23E-46 |
| LOC115594449 | 2.92 | 1.37E-30 |  | LOC115578682 | -2.04 | 4.75E-21 |  | Hr38 | -3.31 | 6.06E-44 |  | DNAJB1 | -3.56 | 1.14E-45 |
| GIMAP5-like | 2.74 | 1.18E-29 |  | LOC115569113 | -2.00 | 1.85E-18 |  | DNAJB1 | -3.20 | 5.69E-40 |  | HBA1 like | -3.15 | 1.24E-45 |
| ZBED1-likr | 4.93 | 1.24E-29 |  | UQCC1 | -2.97 | 2.24E-18 |  | C1QL4-like | 5.65 | 5.49E-39 |  | heme oxygenase like | -4.25 | 1.51E-35 |
| dpy-30 | 3.98 | 1.62E-28 |  | ATRX-like | 1.71 | 6.44E-14 |  | HBA1 | -2.92 | 7.43E-39 |  | HSP90AA1 | -2.65 | 7.10E-34 |
| SAMD9L | 2.42 | 7.20E-28 |  | CSC1-like | 2.13 | 4.75E-11 |  | JUNB | -2.79 | 3.81E-37 |  | HBA1 | -2.50 | 4.13E-30 |

**Supplementary Table 3.** List of common genes between comparisons

| **gene_name** | **gene_description** |
| --- | --- |
| *c23h16orf89* | *chromosome 23C16 orf 89 homolog* |
| *ccnb1* | *cyclin B1* |
| *gadd45g* | *growth arrest and DNA damage inducible gamma* |
| *gdf15* | *growth differentiation factor 15* |
| *gtpbp1* | *GTP binding protein 1%2 C transcript variant X2* |
| *LOC115566982* | *protein asteroid homolog1-like%2C transcript variantX1* |
| *LOC115566997* | *protein NLRC3-like* |
| *LOC115567006* | *hepcidin-like* |
| *LOC115567129* | *protein asteroid homolog1-like%2C transcrip tvariant X1* |
| *LOC115567534* | *uncharacterized LOC115567534* |
| *LOC115568509* | *piggy Bactransposable element-derived protein 3-like%2C transcript variantX1* |
| *LOC115568963* | *coagulation factorVIII-like* |
| *LOC115569649* | *mannose-specificlectin-like%2C transcript variantX1* |
| *LOC115569844* | *histone-lysineN-methyltransferase2C-like* |
| *LOC115570099* | *cystatin-A1-like* |
| *LOC115570105* | *cystatin-A1-like* |
| *LOC115570106* | *cystatin-A1-like%2C transcript variantX3* |
| *LOC115570494* | *membrane-bound ion factor site-2 protease-like* |
| *LOC115570670* | *hemoglobin subunit alpha-1-like* |
| *LOC115570751* | *hemoglobin subunit beta-1-like* |
| *LOC115570826* | *neoverrucotoxin subunit alpha-like* |
| *LOC115571495* | *zinc finger BED domain-containing protein 1-like%2C transcript variantX3* |
| *LOC115572075* | *uncharacterized LOC115572075* |
| *LOC115572813* | *cAMP-responsive element modulator-like%2C transcript variantX3* |
| *LOC115573105* | *tripartite motif-containing protein 16-like* |
| *LOC115573531* | *activator of 90 kDa heatshock protein ATPase homolog1-like%2C transcript variantX1* |
| *LOC115574290* | *heatshock protein HSP90-alpha* |
| *LOC115574332* | *uncharacterized LOC115574332* |
| *LOC115574357* | *cytochrome c oxidase subunit 8A 2C mitochondrial-like* |
| *LOC115575262* | *sterile alpha motif domain-containing protein 9-like* |
| *LOC115575439* | *immunoglobulin lambda-1 light chain-like* |
| *LOC115575516* | *uncharacterized LOC115575516* |
| *LOC115576012* | *histone acetyltransferase KAT7-like %2C transcript variantX3* |
| *LOC115576023* | *heatshock 70 kDa protein 1%2C transcript variantX1* |
| *LOC115576212* | *hemoglobin subunit beta-2-like* |
| *LOC115576218* | *hemoglobin subunit alpha-1* |
| *LOC115576233* | *heatshock 70kDa protein 1-like* |
| *LOC115576265* | *hemeoxygenase-like* |
| *LOC115576604* | *verrucotoxin subunit beta-like%2C transcript variantX3* |
| *LOC115576624* | *NLR family CARD domain-containing protein 3-like* |
| *LOC115577357* | *uncharacterized LOC115577357* |
| *LOC115578591* | *major histocompatibility complex classI-related gene protein -like%2C transcript variantX2* |
| *LOC115578666* | *major histocompatibility complex classI-relatedgene protein -like%2C transcript variantX2* |
| *LOC115578673* | *major histocompatibility complex classI-relatedgene protein -like* |
| *LOC115578726* | *exostosin-1c%2C transcript variantX2* |
| *LOC115578763* | *nucleolar protein of40kDa-like* |
| *LOC115579043* | *major histocompatibility complex classI-related gene protein -like* |
| *LOC115579120* | *sialidase-4-like* |
| *LOC115579676* | *uncharacterized LOC115579676* |
| *LOC115580397* | *myeline xpression factor2-like%2C transcript variantX3* |
| *LOC115580404* | *uncharacterized LOC115580404* |
| *LOC115580668* | *myelin expression factor2-like%2C transcript variantX3* |
| *LOC115581327* | *uncharacterized LOC115581327 %2C transcript variantX3* |
| *LOC115581330* | *centromere-associated protein E-like* |
| *LOC115581728* | *CSC1-like protein 2%2C transcript variantX3* |
| *LOC115582517* | *cytidine deaminase-like* |
| *LOC115583117* | *interferon-induced GTP-binding protein Mx-like* |
| *LOC115583216* | *NLR family CARD domain-containing protein 3-like* |
| *LOC115583218* | *NLR family CARD domain-containing protein 3-like* |
| *LOC115583240* | *nuclear factor 7%2C brain-like* |
| *LOC115583258* | *deoxyribonuclease-1-like* |
| *LOC115583363* | *probable nuclear hormone receptor HR38* |
| *LOC115583916* | *protein dpy-30 homolog* |
| *LOC115583929* | *ubiquinol-cytochrome-c reductase complex assembly factor1-like* |
| *LOC115584526* | *dna J homolog subfamily B member1-like* |
| *LOC115585098* | *deoxynucleoside triphosphate triphosphohydrolase SAMHD1-like* |
| *LOC115585644* | *sodium-and chloride-dependent creatine transporter1-like* |
| *LOC115585833* | *interferon-inducible GTPase 5-like* |
| *LOC115586307* | *homeo domain-interacting protein kinase3-like%2C transcript variantX1* |
| *LOC115587065* | *probable E3 ubiquitin - protein ligase TRIML1* |
| *LOC115587597* | *interferon-induced protein 44-like* |
| *LOC115587647* | *interferon-induced protein 44-like* |
| *LOC115587823* | *uncharacterized LOC115587823* |
| *LOC115587828* | *golgin subfamily A member 6-like protein 22%2C transcript variantX1* |
| *LOC115587986* | *transcript ion factor jun-B-like%2C transcript variantX2* |
| *LOC115588483* | *hemeoxygenase-like* |
| *LOC115588739* | *GTPase IMAP family member 5-like* |
| *LOC115588751* | *interferon-induced protein 44-like* |
| *LOC115589114* | *complement C1q-like protein 4* |
| *LOC115589135* | *myelin-oligodendrocyteglyco protein -like* |
| *LOC115589162* | *uncharacterized LOC115589162* |
| *LOC115589169* | *transcript ionalregulatorATRX-like* |
| *LOC115589597* | *complement C1q-like protein 4* |
| *LOC115589598* | *complement C1q-like protein 3* |
| *LOC115589599* | *complement C1q-like protein 4* |
| *LOC115589600* | *complement C1q-like protein 4* |
| *LOC115589661* | *complement C1q-like protein 4* |
| *LOC115590011* | *uncharacterized LOC115590011* |
| *LOC115590037* | *uncharacterized LOC115590037* |
| *LOC115590048* | *uncharacterized LOC115590048* |
| *LOC115590073* | *hepatitis A virus cellular receptor 2* |
| *LOC115590198* | *complement C1q-like protein 4* |
| *LOC115590271* | *E3 ubiquitin - protein ligaseTRIM39-like* |
| *LOC115590329* | *uncharacterized LOC115590329* |
| *LOC115590507* | *GTPase IMAP famil ymember 7-like %2C transcript variantX1* |
| *LOC115590564* | *ubiquitin carboxyl-terminalhydrolase47-like* |
| *LOC115590941* | *receptor-transporting protein 3-like%2C transcript variantX3* |
| *LOC115590992* | *lysine-specific demethylase 4 A-like* |
| *LOC115591912* | *hydroxylysine kinase-like%2C transcript variantX4* |
| *LOC115591945* | *FRAS1-related extracellular matrix protein 1-like* |
| *LOC115592083* | *E3 ubiquitin - protein ligase RNF14-like%2C transcript variantX1* |
| *LOC115592192* | *uncharacterized LOC115592192 %2C transcript variantX2* |
| *LOC115592674* | *uncharacterized LOC115592674* |
| *LOC115592678* | *uncharacterized LOC115592678* |
| *LOC115592721* | *uncharacterized LOC115592721* |
| *LOC115592912* | *uncharacterized LOC115592912 %2C transcript variantX2* |
| *LOC115593050* | *heatshock protein 30-like* |
| *LOC115593735* | *uncharacterized LOC115593735* |
| *LOC115594786* | *hexokinase-2-like* |
| *LOC115595589* | *23 kDa integralmembrane protein -like* |
| *LOC115596200* | *cytochrome c oxidase subunit 7A-related protein %2Cmitochondrial-like* |
| *LOC115596834* | *echinoderm microtubule-associated protein -like6* |
| *meiob* | *meiosis specific with OB-fold%2C transcript variantX3* |
| *nr4a3* | *nuclear receptor subfamily 4 group A member3%2C transcript variantX1* |
| *plk1* | *polo like kinase 1%2 C transcript variant X2* |
| *rgs7* | *regulator of G protein signaling 7%2C transcript variant X3* |
| *samd3* | *sterile alpha motif domain containing 3%2C transcript variant X3* |
| *wnk4* | *WNK lysine deficient protein kinase 4%2C transcript variant X3* |


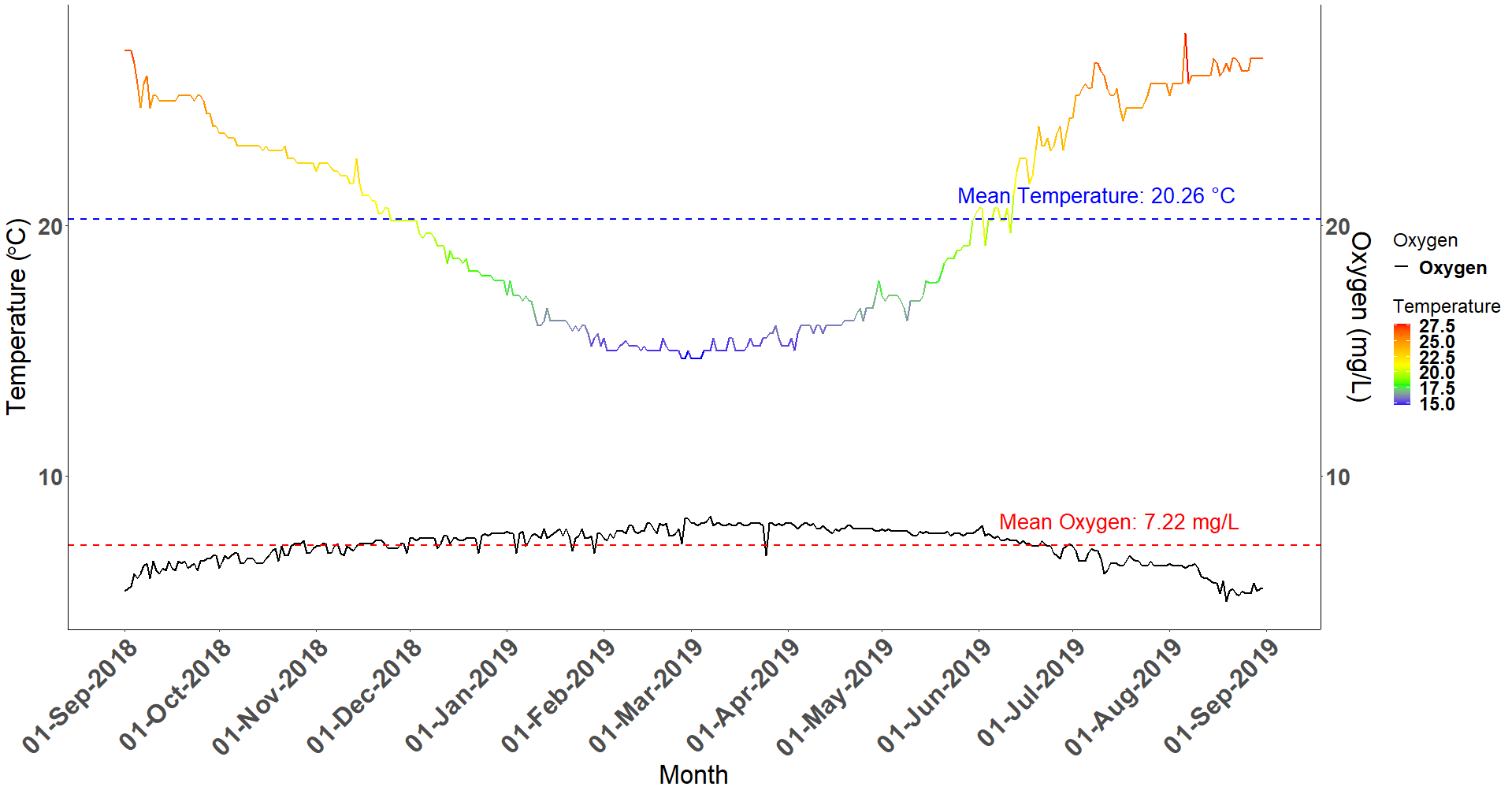


**Supplementary Figure 1.** Sea temperature variation throughout the feeding trial. Color depicts the alteration of temperature from the mean value (20.26 ^o^C) and oxygen levels (black line), with dashed lines indicating their respective mean values.


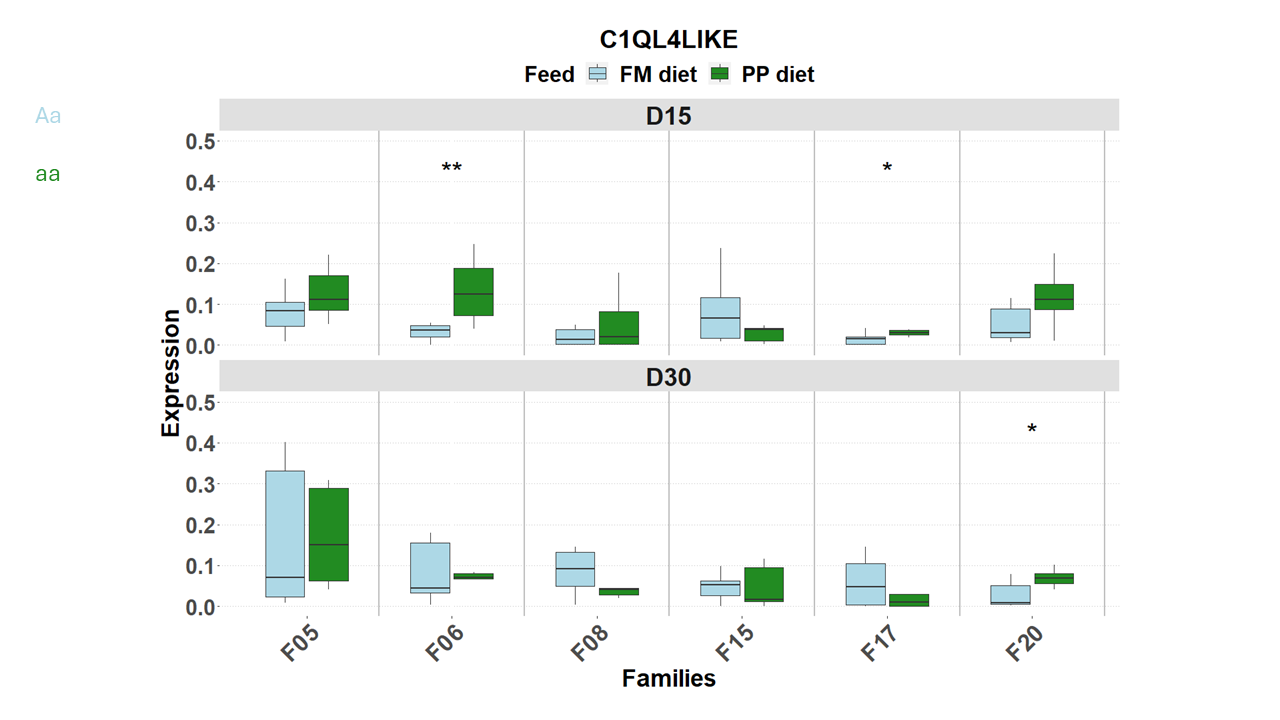


**Supplementary Figure 2.** Gene expression of C1QL4 in the erythrocytes per family, 15 and 30 days after trial initiation. Significant changes between feeds (light blue: FM diet, green: PP diet) are marked with asterisks (∗). Significance levels are presented on the plot (*<0.05, **<0.01, ***<0.001, ***<0.0001).

**
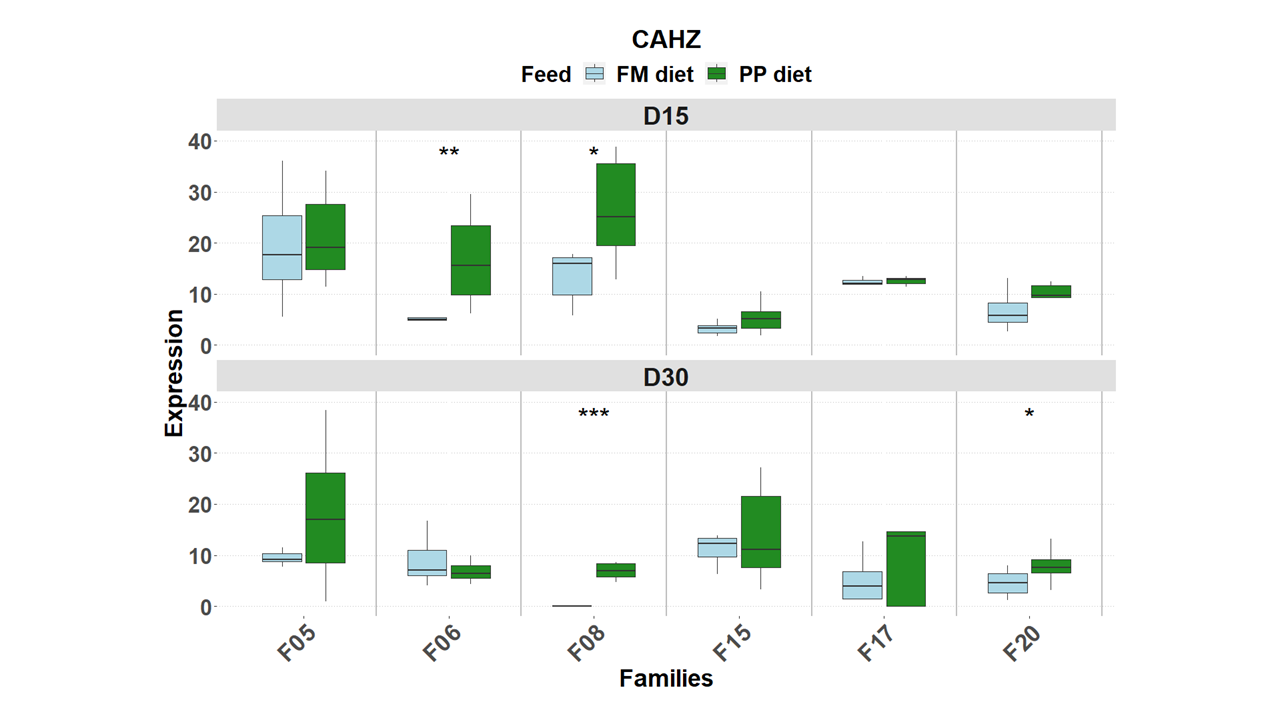
**

**Supplementary Figure 3.** Gene expression of CAHZ in the erythrocytes per family, 15 and 30 days after trial initiation. Significant changes between feeds (light blue: FM diet, green: PP diet) are marked with asterisks (∗). Significance levels are presented on the plot (*<0.05, **<0.01, ***<0.001, ***<0.0001).

**
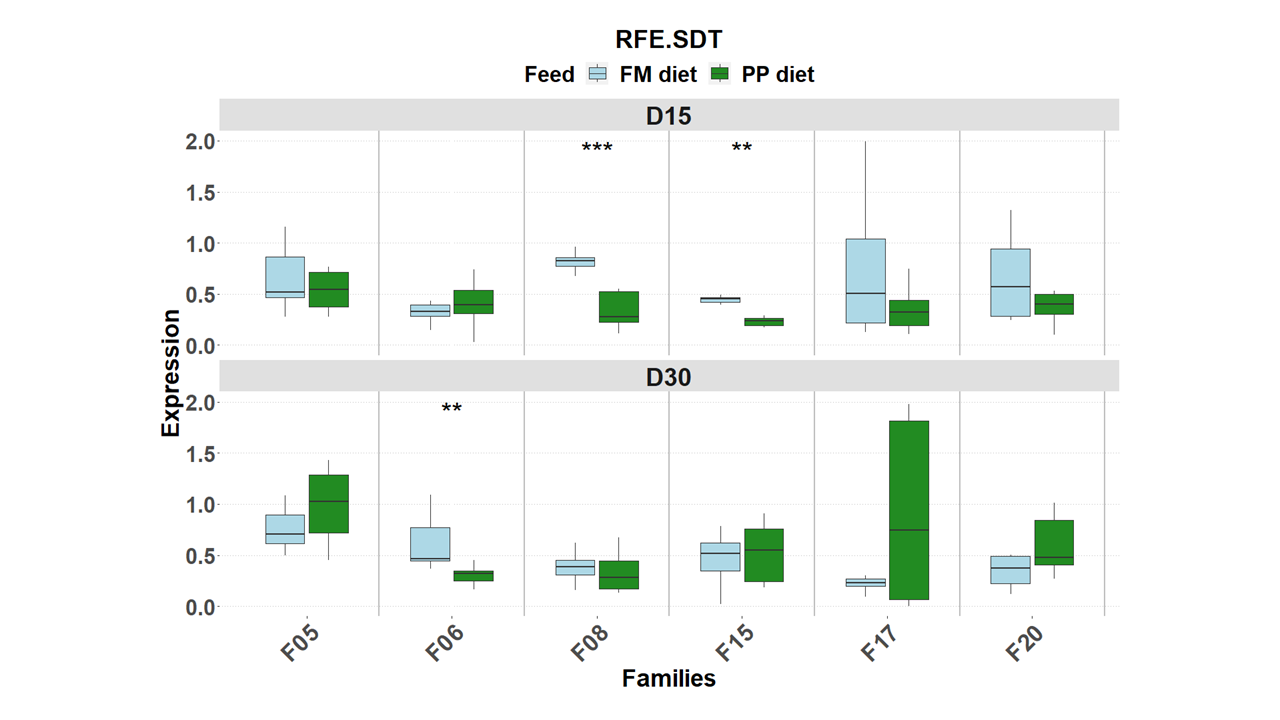
**

**Supplementary Figure 4.** Gene expression of rfe.sdt in the erythrocytes per family, 15 and 30 days after trial initiation. Significant changes between feeds (light blue: FM diet, green: PP diet) are marked with asterisks (∗). Significance levels are presented on the plot (*<0.05, **<0.01, ***<0.001, ***<0.0001).

**
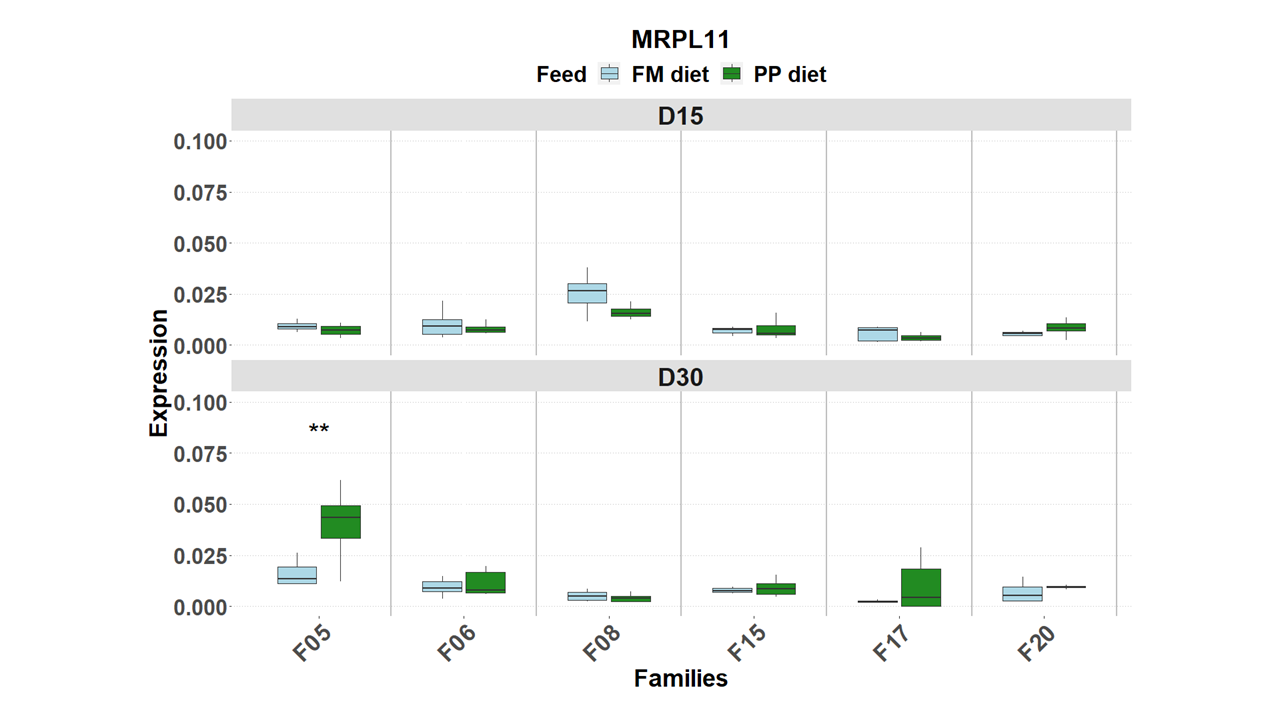
**

**Supplementary Figure 5.** Gene expression of mrpl11 in the erythrocytes per family, 15 and 30 days after trial initiation. Significant changes between feeds (light blue: FM diet, green: PP diet) are marked with asterisks (∗). Significance levels are presented on the plot (*<0.05, **<0.01, ***<0.001, ***<0.0001).

**
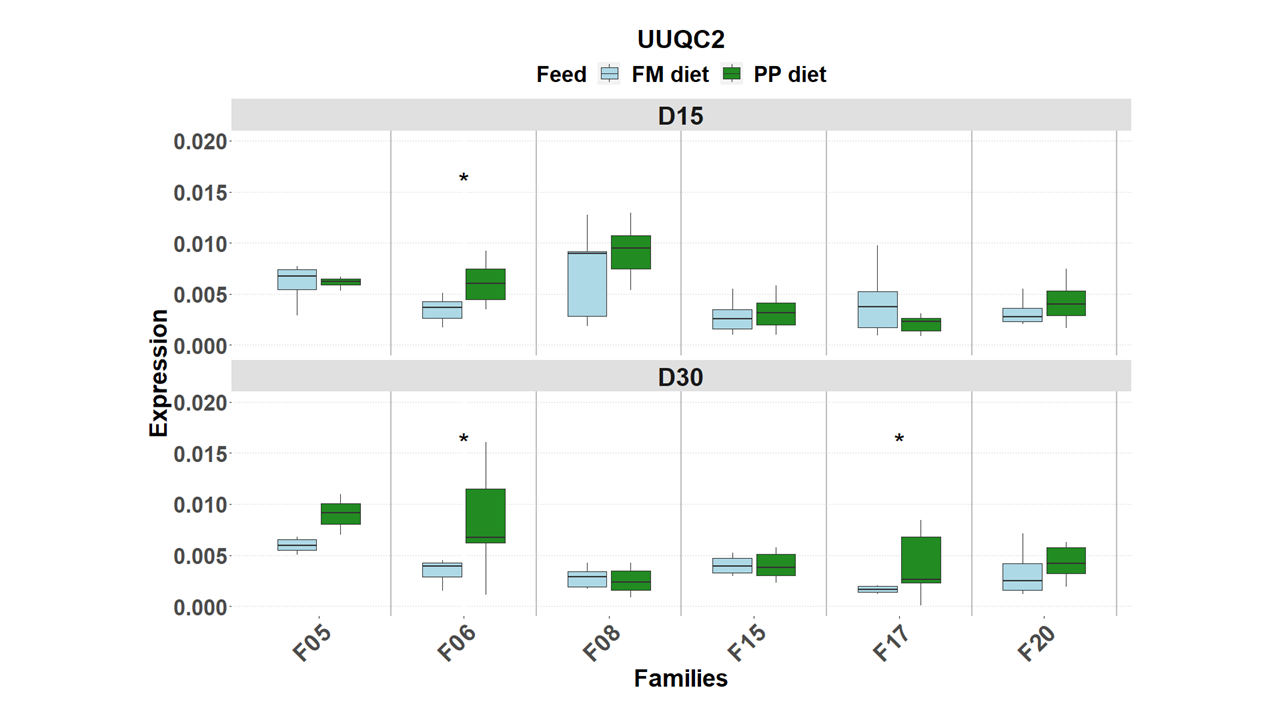
**

**Supplementary Figure 6.** Gene expression of uuqc2 in the erythrocytes per family, 15 and 30 days after trial initiation. Significant changes between feeds (light blue: FM diet, green: PP diet) are marked with asterisks (∗). Significance levels are presented on the plot (*<0.05, **<0.01, ***<0.001, ***<0.0001).

**
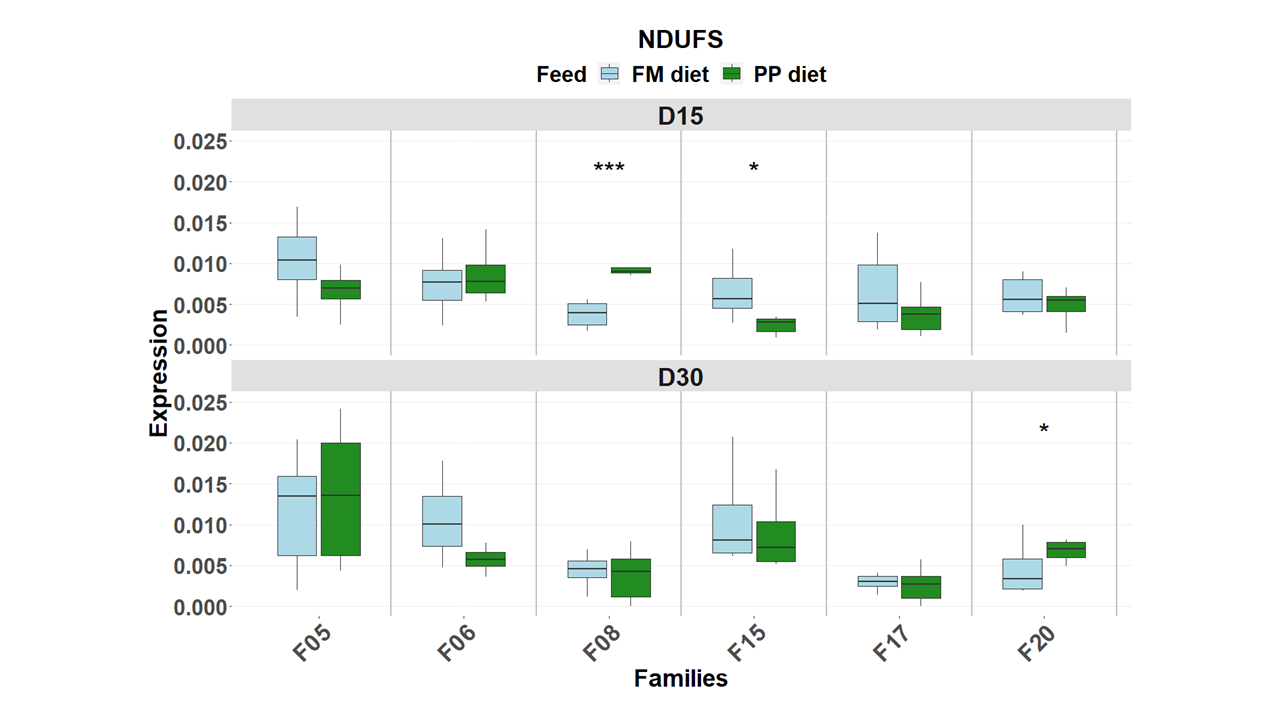
**

**Supplementary Figure 7.** Gene expression of ndufs in the erythrocytes per family, 15 and 30 days after trial initiation. Significant changes between feeds (light blue: FM diet, green: PP diet) are marked with asterisks (∗). Significance levels are presented on the plot (*<0.05, **<0.01, ***<0.001, ***<0.0001).

**
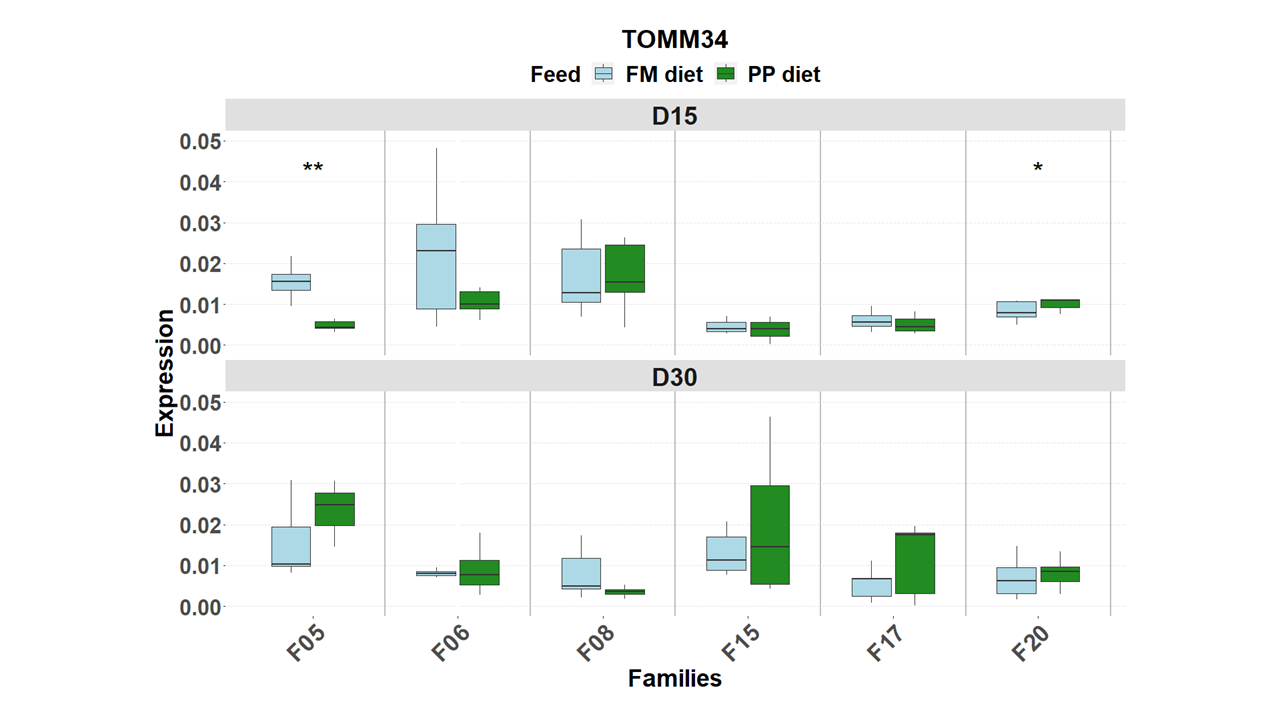
**

**Supplementary Figure 8.** Gene expression of tomm34 in the erythrocytes per family, 15 and 30 days after trial initiation. Significant changes between feeds (light blue: FM diet, green: PP diet) are marked with asterisks (∗). Significance levels are presented on the plot (*<0.05, **<0.01, ***<0.001, ***<0.0001).

**
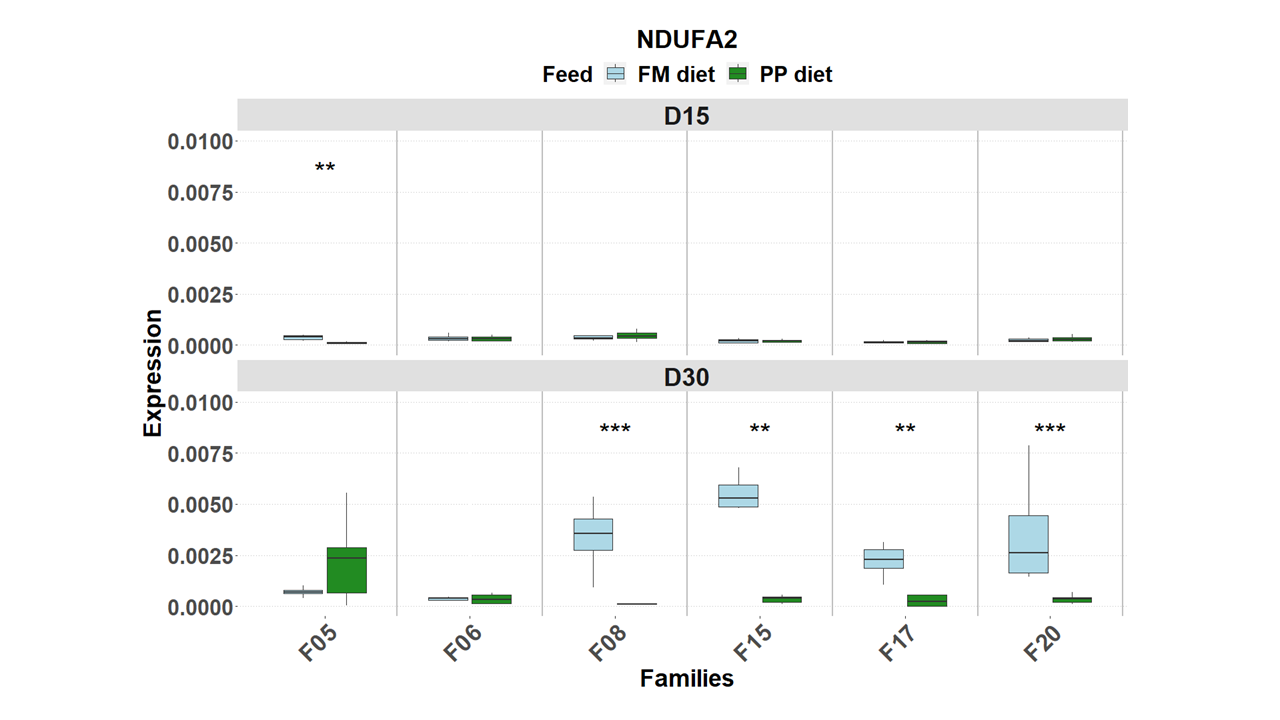
**

**Supplementary Figure 9.** Gene expression of ndufa2 in the erythrocytes per family, 15 and 30 days after trial initiation. Significant changes between feeds (light blue: FM diet, green: PP diet) are marked with asterisks (∗). Significance levels are presented on the plot (*<0.05, **<0.01, ***<0.001, ***<0.0001).

**
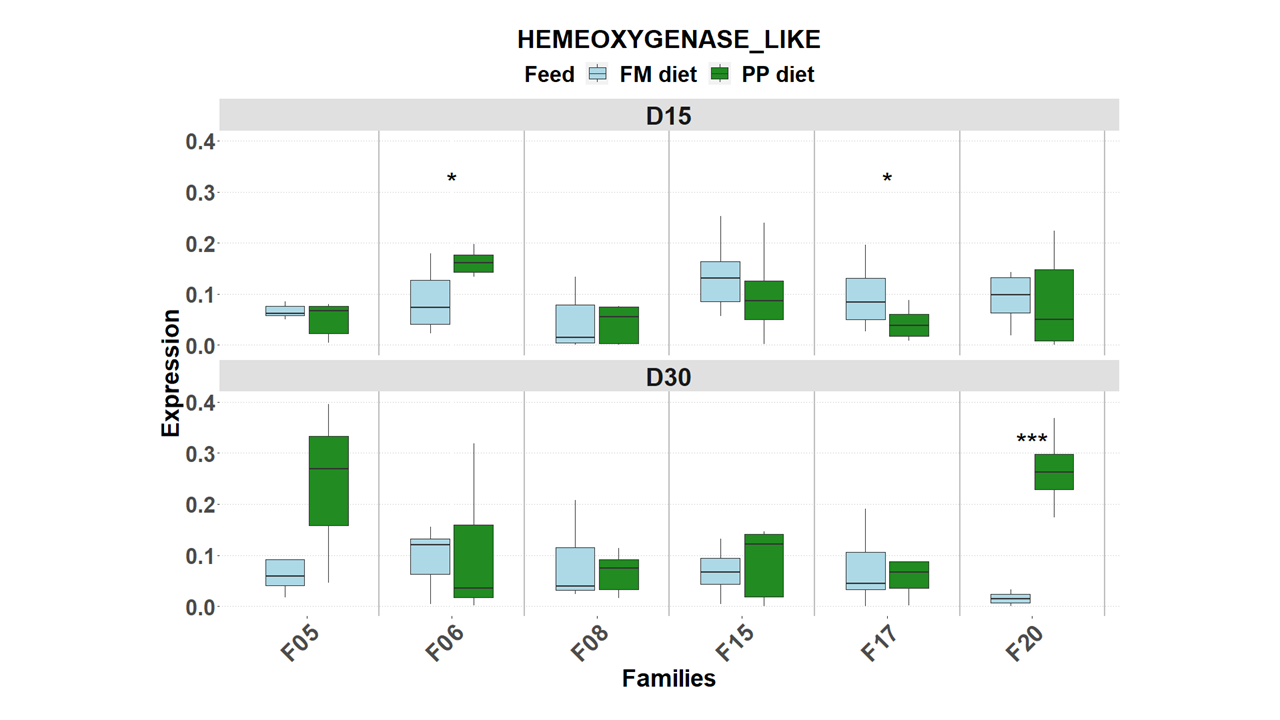
**

**Supplementary Figure 10.** Gene expression of hmo2 in the erythrocytes per family, 15 and 30 days after trial initiation. Significant changes between feeds (light blue: FM diet, green: PP diet) are marked with asterisks (∗). Significance levels are presented on the plot (*<0.05, **<0.01, ***<0.001, ***<0.0001).

**
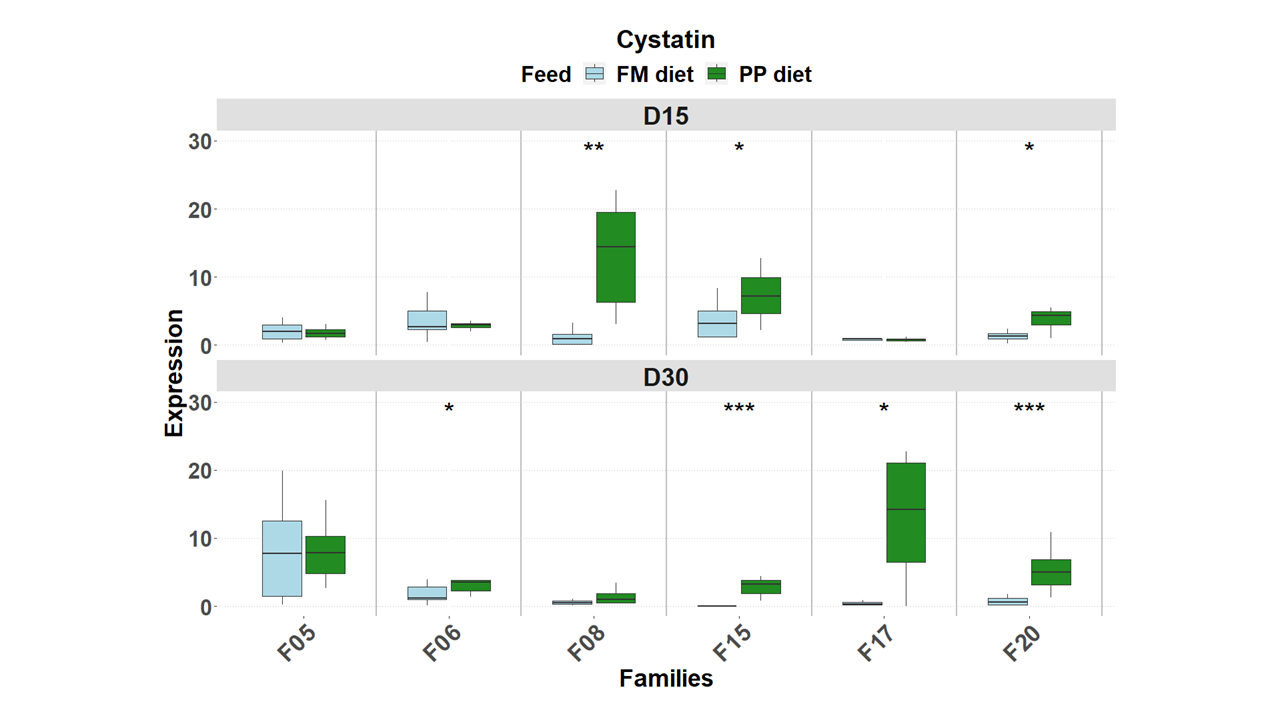
**

**Supplementary Figure 11.** Gene expression of cystatin in the erythrocytes per family, 15 and 30 days after trial initiation. Significant changes between feeds (light blue: FM diet, green: PP diet) are marked with asterisks (∗). Significance levels are presented on the plot (*<0.05, **<0.01, ***<0.001, ***<0.0001).

**
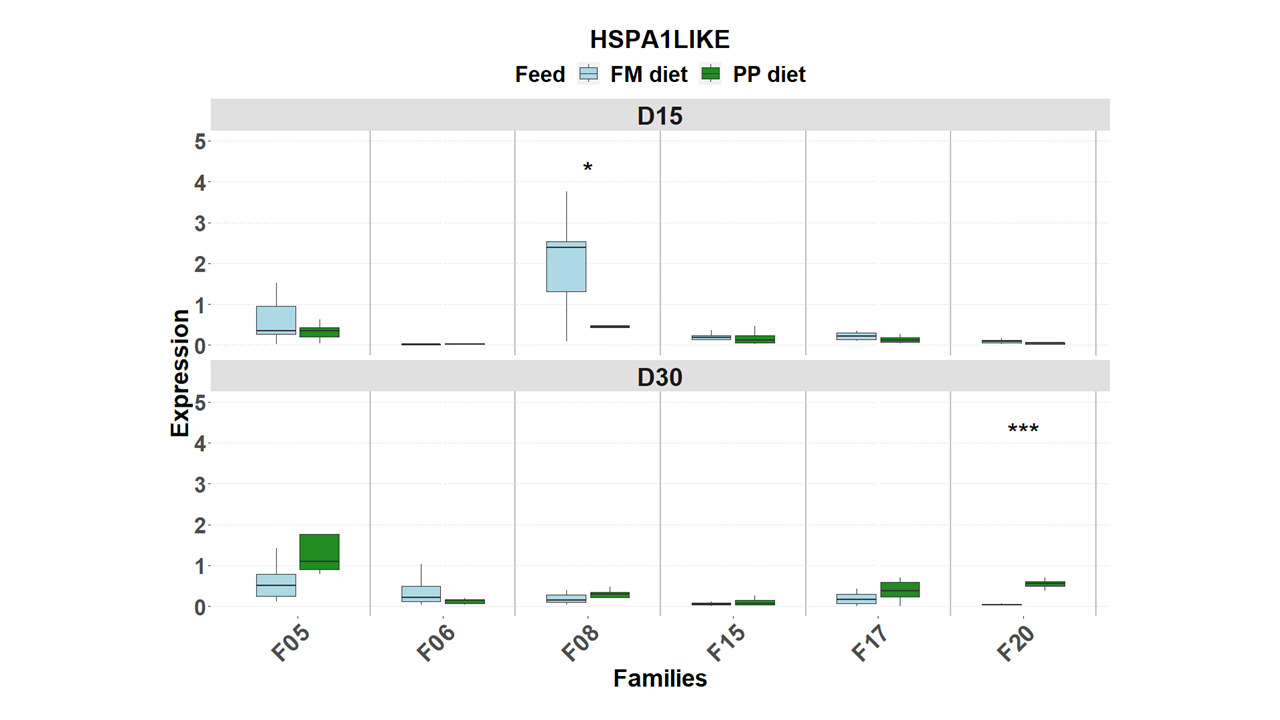
**

**Supplementary Figure 12.** Gene expression of hspa1l in the erythrocytes per family, 15 and 30 days after trial initiation. Significant changes between feeds (light blue: FM diet, green: PP diet) are marked with asterisks (∗). Significance levels are presented on the plot (*<0.05, **<0.01, ***<0.001, ***<0.0001).

**
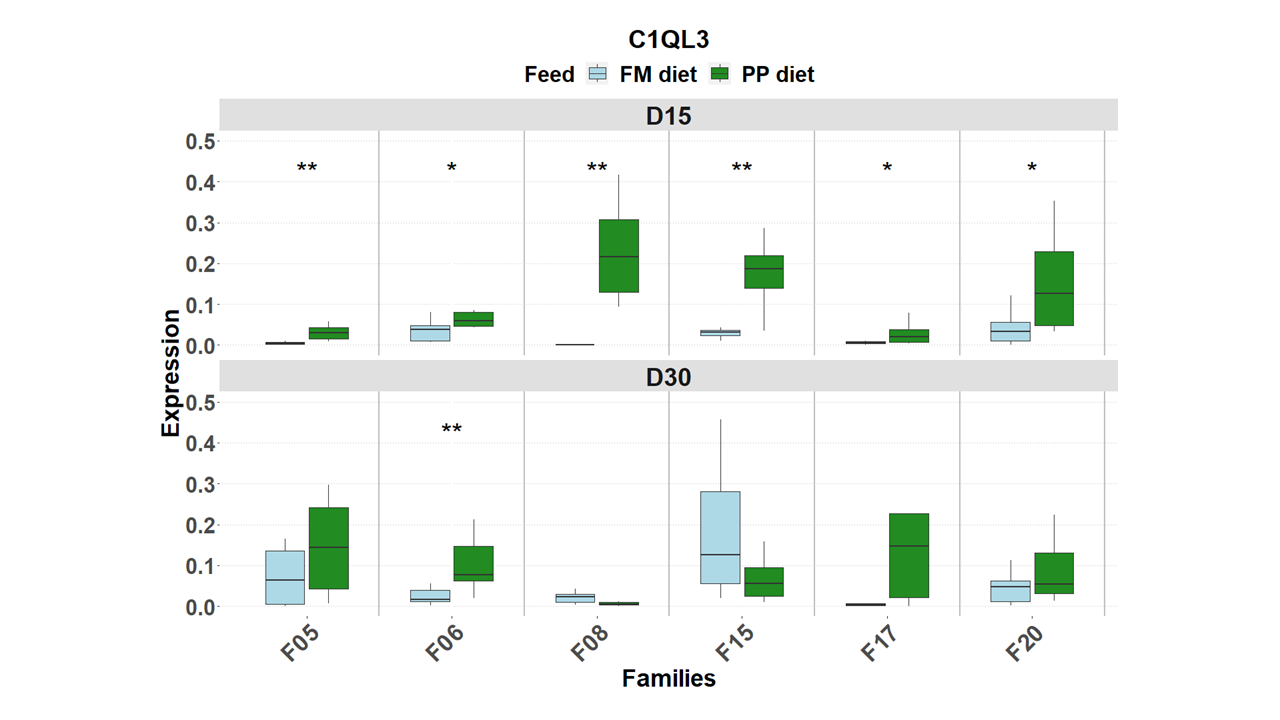
**

**Supplementary Figure 13.** Gene expression of c1ql3 in the erythrocytes per family, 15 and 30 days after trial initiation. Significant changes between feeds (light blue: FM diet, green: PP diet) are marked with asterisks (∗). Significance levels are presented on the plot (*<0.05, **<0.01, ***<0.001, ***<0.0001).

**
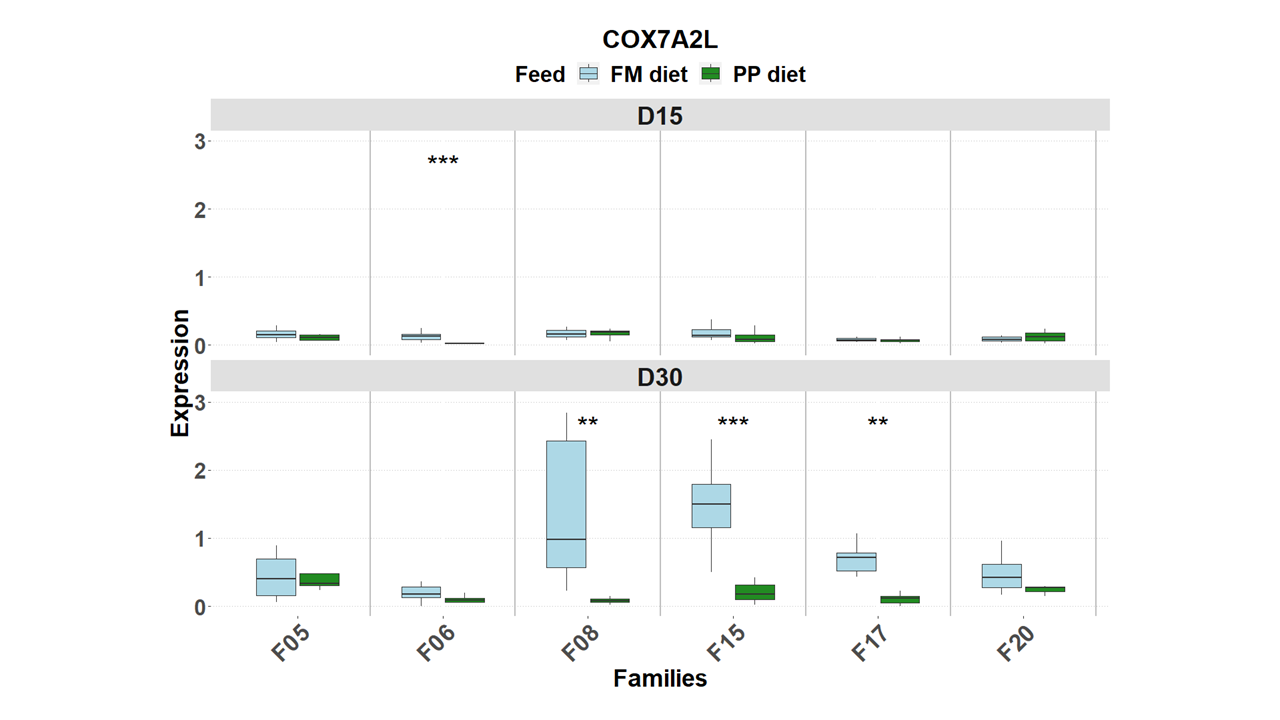
**

**Supplementary Figure 14.** Gene expression of cox7a2l in the erythrocytes per family, 15 and 30 days after trial initiation. Significant changes between feeds (light blue: FM diet, green: PP diet) are marked with asterisks (∗). Significance levels are presented on the plot (*<0.05, **<0.01, ***<0.001, ***<0.0001).

**
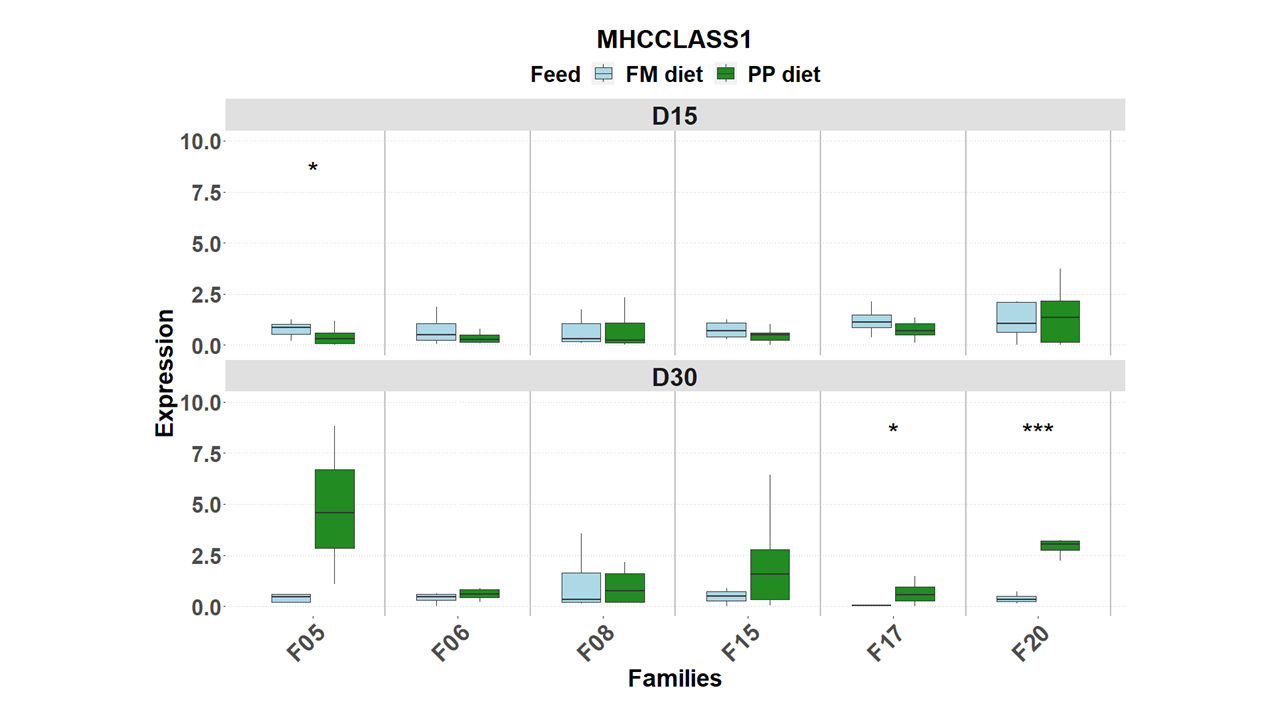
**

**Supplementary Figure 15.** Gene expression of MHC class 1 in the erythrocytes per family, 15 and 30 days after trial initiation. Significant changes between feeds (light blue: FM diet, green: PP diet) are marked with asterisks (∗). Significance levels are presented on the plot (*<0.05, **<0.01, ***<0.001, ***<0.0001).

**
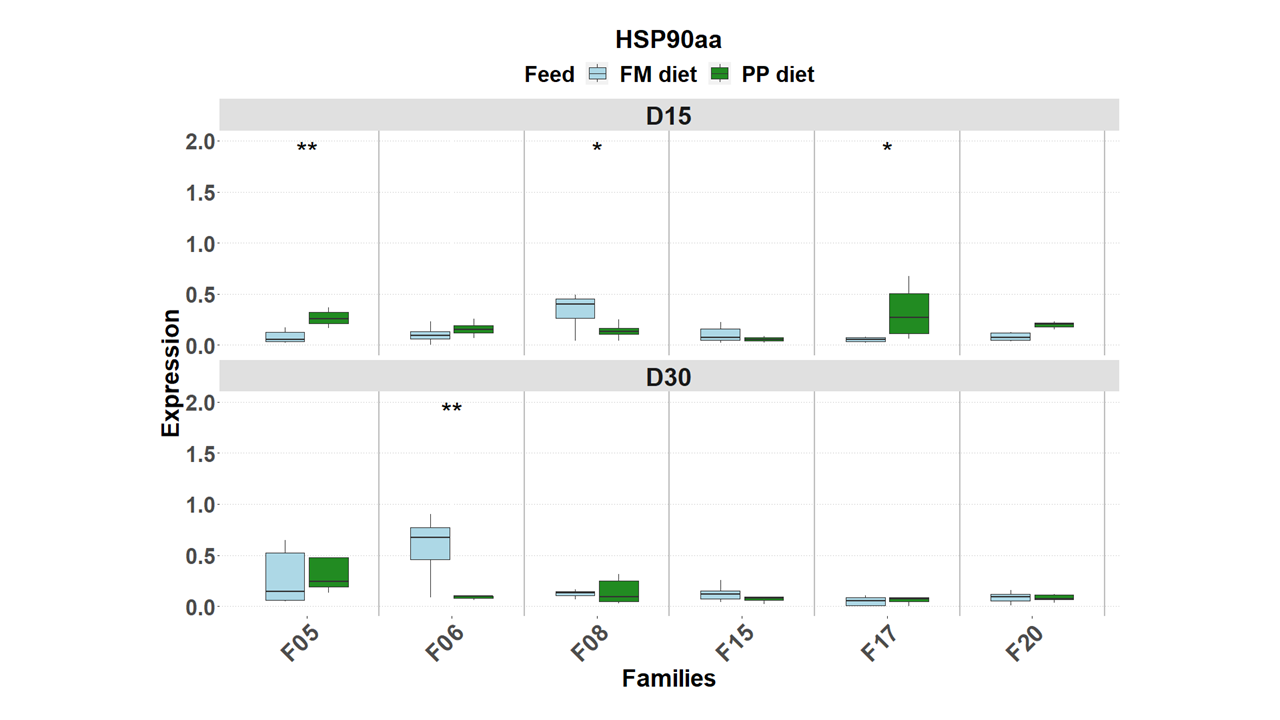
**

**Supplementary Figure 16.** Gene expression of hsp90aa in the erythrocytes per family, 15 and 30 days after trial initiation. Significant changes between feeds (light blue: FM diet, green: PP diet) are marked with asterisks (∗). Significance levels are presented on the plot (*<0.05, **<0.01, ***<0.001, ***<0.0001).

**
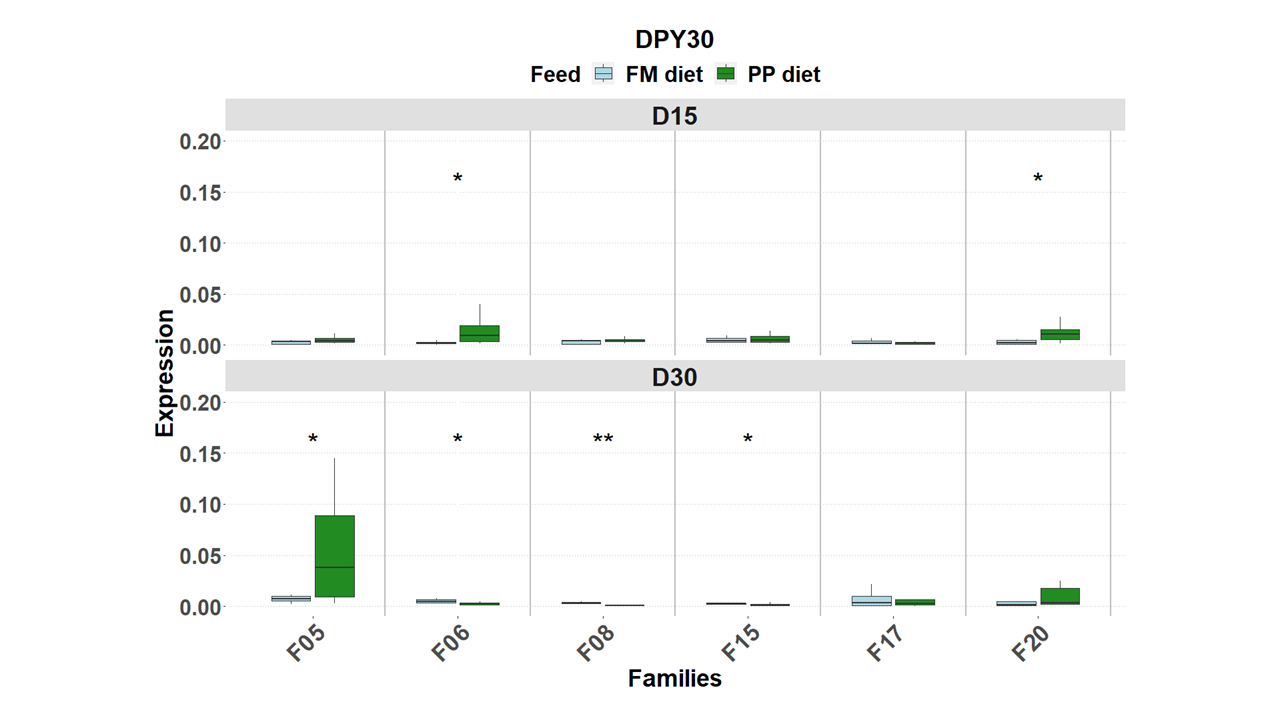
**

**Supplementary Figure 17.** Gene expression of dpy30 in the erythrocytes per family, 15 and 30 days after trial initiation. Significant changes between feeds (light blue: FM diet, green: PP diet) are marked with asterisks (∗). Significance levels are presented on the plot (*<0.05, **<0.01, ***<0.001, ***<0.0001).
